# Supplementary material for: Integration of Geriatrics and Palliative Medicine Into a Medical Student Clinical Reasoning Curriculum
Source: MedEdPORTAL. 2025 Feb 6;21:11495. doi: 10.15766/mep_2374-8265.11495 (PMC11799358; doi:10.15766/mep_2374-8265.11495)
Supplement: Supplementary file 1 — Facilitator Guide.docxPhysical Exam Findings.pptxStudent Survey.docxFaculty Survey.docx [file mep_2374-8265.11495-s001.zip › C. Student Survey.docx]

**Student Survey**

For each of the topics listed below, please check the box that best that indicates your level of confidence both **before** and **after** today’s Clinical Learning Session:

**1 = Not Confident 2 = Slightly Confident 3 = Moderately Confident 4 = Confident 5 = Very Confident**

|  | **Confidence BEFORE the session** | | | | |  | **Confidence AFTER the session** | | | | |
| --- | --- | --- | --- | --- | --- | --- | --- | --- | --- | --- | --- |
| **How do you rate your confidence about the following topics?** | **1** | **2** | **3** | **4** | **5** |  | **1** | **2** | **3** | **4** | **5** |
| Applying your communication skills in the care of an older adult patient presenting with a change in mental status |  |  |  |  |  |  |  |  |  |  |  |
| Applying your clinical reasoning skills in the care of an older adult patient presenting with a change in mental status |  |  |  |  |  |  |  |  |  |  |  |
| Applying your hypothesis-driven physical exam skills in the care of an older adult patient presenting with a change in mental status |  |  |  |  |  |  |  |  |  |  |  |
| Gathering a history from a patient’s family member. |  |  |  |  |  |  |  |  |  |  |  |
| Considering a patient’s goals of care in developing a diagnostic and treatment plan for an older adult presenting with a change in mental status. |  |  |  |  |  |  |  |  |  |  |  |

| I found the integration of geriatric and palliative medicine concepts into today’s CLS case an effective means of applying prior knowledge. | Strongly  Disagree | Disagree | Agree | Strongly Agree |
| --- | --- | --- | --- | --- |

Please share your thoughts, observations, and/or reactions to the integration of the patient’s MOLST form in the evaluation and management discussion of today’s CLS case:
