# Supplementary material for: Integration of Geriatrics and Palliative Medicine Into a Medical Student Clinical Reasoning Curriculum
Source: MedEdPORTAL. 2025 Feb 6;21:11495. doi: 10.15766/mep_2374-8265.11495 (PMC11799358; doi:10.15766/mep_2374-8265.11495)
Supplement: Supplementary file 1 — Facilitator Guide.docxPhysical Exam Findings.pptxStudent Survey.docxFaculty Survey.docx [file mep_2374-8265.11495-s001.zip › D. Faculty Survey.docx]

**Faculty Survey**

| **During today’s session, how effectively were the following learning objectives accomplished:** | **Not at all effectively** | **A little bit effectively** | **Somewhat effectively** | **Very effectively** | **Extremely effectively** |
| --- | --- | --- | --- | --- | --- |
| Learners were able to apply their communication skills in the care of an older adult patient presenting with a change in mental status |  |  |  |  |  |
| Learners were able to apply their clinical reasoning skills in the care of an older adult patient presenting with a change in mental status |  |  |  |  |  |
| Learners were able to apply their physical diagnosis skills in the care of an older adult patient presenting with a change in mental status |  |  |  |  |  |
| Learners were able to gather a history from a patient’s family member |  |  |  |  |  |
| Learners were able to consider a patient’s goals of care in developing a diagnostic and treatment plan for an older adult presenting with a change in mental status. |  |  |  |  |  |

| I found the integration of geriatric and palliative medicine concepts into today’s CLS case an effective means of allowing students to apply prior knowledge. | Strongly  Disagree | Disagree | Agree | Strongly Agree |
| --- | --- | --- | --- | --- |

Please share your thoughts, observations, and reactions to the integration of the patient’s MOLST form in the evaluation and management discussion of today’s CLS case:
