# Supplementary material for: Integration of Geriatrics and Palliative Medicine Into a Medical Student Clinical Reasoning Curriculum
Source: MedEdPORTAL. 2025 Feb 6;21:11495. doi: 10.15766/mep_2374-8265.11495 (PMC11799358; doi:10.15766/mep_2374-8265.11495)
Supplement: Supplementary file 1 — Facilitator Guide.docxPhysical Exam Findings.pptxStudent Survey.docxFaculty Survey.docx [file mep_2374-8265.11495-s001.zip › A. Facilitator Guide.docx]

**Clinical Learning Session: Change in Mental Status**

**Facilitator Guide**

**Set up and Logistics:**

This is a 110-minute small group session designed to take place in a classroom setting with pre-clerkship medical students and two faculty facilitators.

*Classroom Suggestions*

1. Room with seating, ideally seminar table so that students can all sit around one table with ability of the students to move to sit near faculty when they are gathering the patient history
2. Media configuration: ability to display PowerPoint, whiteboard or chalkboard and appropriate writing implements

**Clinical Learning Curriculum Goals:**

*By the end of the curriculum students will be able to:*

1. Clinical Reasoning
   1. Generate an organ system/ pathophysiologic-based differential for common clinical concerns based on a patient’s history.
   2. Compose an accurate problem representation using semantic qualifiers from information collected in an initial patient encounter.
   3. Apply one or more illness scripts to match the patient they are evaluating.
2. Hypothesis Driven Physical Diagnosis
   1. Identify, justify, and complete a focused physical exam based upon their differential diagnosis.
   2. Interpret physical examination findings.
3. Oral Presentation
   1. Present a patient’s history of present illness (HPI) inclusive of a clear opening statement, appropriate pertinent positive/negative findings, and semantic qualifiers.

**Case-Specific Learning Objectives:**

*By the end of this activity, learners will be able to:*

1. Apply their communication, clinical reasoning, and hypothesis-driven physical diagnosis skills in the care of an older adult presenting with a change in mental status.
2. Practice gathering a history from a patient’s family member.
3. Consider a patient’s goals of care in developing a diagnostic and treatment plan for an older adult presenting with a change in mental status.

**Faculty Pre-Work**

1. Review Faculty Guide and PowerPoint in advance of session

**Faculty Development Pre-meeting Agenda (30 minutes total)**

This section of the guide is intended for use by the faculty directing the CLS Change in Mental Status pre-meeting. The goal of the premeeting is to maximize consistency across the small groups.

Faculty Development Meeting outline with questions for facilitation:

1. Review the clinical case and answer faculty questions about the case content (5 minutes)
   1. *What questions do you have about the clinical case?*
   2. Review the 4Ms Framework for the care of the Older Adult (What Matters Most, Medication, Mentation, Mobility)
   3. Highlight use of hyperlinks in the PowerPoint and review the MOLST/Advance Care Plan
2. Contextualize the learners for faculty who do not regularly work with the students (5 minutes)
   1. Review:
      - Learner level of training
      - Learner past exposure to principles of geriatrics and palliative medicine
      - Learner past exposure to principles of pharmacology, neurology, psychiatry
3. Review and reinforce the empathic opportunities present in the case to promote authentic portrayal and ensure opportunity to use empathy skills to build rapport and trust and to further explore the current presentation. (5 minutes)
4. Discuss the importance of the students identifying an organizational framework for their hypothesis-generation. (5 minutes)
   1. *What has worked in the past for learners at this level? Body System vs Mechanistic Approach?*
5. Provide time for faculty partners to meet and strategize their approach to facilitation to maximize learner participation. (10 minutes)
   1. Questions for faculty to address
      1. *Who will be playing the role of the patient?*
      2. *How will they work together to facilitate learning and avoid teaching content?*

**Session Outline (****110 minutes total)**

1. Intro and Assignment of Student Roles (5 minutes)
2. History Gathering and Facilitated Discussion (60 minutes)
3. Physical Diagnosis Hypothesis Generation (10 minutes)
4. Group Discussion (10 minutes)
5. Oral Presentation Practice (20 minutes)
6. Wrap-up (5 minutes)

**Intro and Assignment of Student Roles (5 minutes)**

Faculty will facilitate a group introduction including an ice breaker if the students have not previously worked together. Faculty should facilitate a brief discussion about the use of role-play to gather a patient history, normalizing potential discomfort students might feel when gathering portions of the history in front of colleagues. Faculty should encourage students to create ground rules that will improve comfort during the session. Ground rules may include: agreement that what occurs in the session in regard to individual student performance and feedback should remain confidential, encouragement to take risks, encouragement of students to “time out” during role play when they are unsure on how to proceed, commitment that peers will provide feedback that is reinforcing on what was done effectively and will provide specific constructive feedback on areas of uncertainty that have been identified.

*Have students assign themselves to the following roles for the session:*

1. Scribe – one student will be responsible for taking notes on the whiteboard or shared computer document as the history is being gathered.
2. Timeline scribe – one student will be responsible for mapping events gathered from the history on a timeline from point of initial symptoms through today. If not previously discussed, faculty should remind the students of the importance of gathering and organizing the history in a chronological fashion. This will aid in the diagnostic process and in the development of effective oral and written presentations.
3. Timekeeper – this student will remind the group of the time at 15-minute intervals.

**History Gathering and Facilitated Discussion (60 minutes):**

Students will gather a history from a faculty member playing the patient’s adult child. This will be done as a rolling role play, with each student taking a turn to gather portions of the history. The rest of the group will observe and be asked to provide reinforcing feedback to their peers. The students assigned as scribe and timeline scribe should be documenting on the board as the history is being gathered. The group will pause frequently to discuss diagnostic hypotheses, and faculty will facilitate a discussion around the group’s clinical reasoning. Potential diagnostic hypotheses organized by body system are included in Table 1. Students should be encouraged to use their hypotheses to guide the history gathering**.** At the conclusion of the history gathering, the group will work together to develop a problem representation.

1. **Pause frequently during history** **gathering to allow for the group to generate a broad yet plausible list of diagnostic hypotheses and to provide feedback to one another on the use of their communications skills.**
   1. The first pause should be immediately after elicitation of chief concern, at which point the group can discuss:
      1. *What was the patient’s chief concern? What is the patient’s problem around which we need to develop hypotheses?*
      2. *What are some potential causes of this problem?*
   2. Students should be encouraged to make use of the whiteboard in the room to brainstorm potential diagnostic hypotheses. They should organize their hypotheses either by body system or by mechanism.
   3. With each subsequent pause, faculty should:
      1. Ask the student who has been gathering the history: *How is it going? What have you done effectively?*
      2. Ask permission to ask peers for additional feedback on what the student has done effectively, and once permission is granted, elicit reinforcing feedback from the group.
      3. Ask the group:
         1. *What have we learned?*
         2. *How does this information change our hypotheses?*
         3. *Is there anything that you would want to add?*
         4. *Are there any hypotheses that are now more or less likely?*
         5. *How should we use our remaining hypotheses to guide our history gathering? (e.g., consideration of illness scripts – what is the story of this disease, what are the expected associated symptoms we should be eliciting?)*
         6. If additional prompting is necessary: *Ask students if there are any questions from their review of systems list that will help them in further understanding what might be going on?*

| **TABLE 1. Potential Diagnostic Hypotheses for Older Adult with Change in Mental Status** | | |
| --- | --- | --- |
| **Category** | **Possible Etiology** | **Relevant Demographics, Risk Factors, Symptoms and Signs** |
| **Pulmonary** | Infection, see below |  |
|  | Hypoxia/Hypercarbia | Would be higher on list of consideration for patient with history of tobacco smoking or COPD |
| **Cardiovascular** | Myocardial Infarction | Older patients may have atypical including syncope, weakness, or confusion |
|  | Heart Failure | Encephalopathy due to low perfusion state/decreased cardiac output |
|  | Arrhythmia | Encephalopathy due to low perfusion state/ decreased cardiac output |
|  | Hypertensive Emergency | Symptoms include headache, confusion, nausea/vomiting. |
| **Gastroenterology** | Constipation/Fecal Impaction | Constipation is common in older adults, may be due to primary colorectal dysfunction, medication effects, IBS, immobility, or dehydration |
|  | Liver Failure/Hepatic Encephalopathy | It would be important to ask about alcohol use and risk factors for viral hepatitis. May have jaundice/scleral icterus or asterixis on physical exam. |
| **Renal/GU/GYN** | Urinary Retention | May occur from a urinary tract infection, anticholinergic side effects of medications, (benign prostatic hypertrophy is a common reason for urinary retention in men) |
|  | Renal Failure/Uremia | Clinical features of uremic encephalopathy include lethargy, irritability, disorientation, hallucinations, and rambling speech. Tremor, myoclonus, and asterixis may be seen on the physical exam. May also have signs and symptoms of volume overload on history and exam. |
| **Hematologic/**  **Oncologic** | CNS tumor/space occupying lesion | May have focal neurologic deficit or seizure |
|  | Paraneoplastic syndrome | Paraneoplastic neurologic syndromes may affect any part of the nervous system, from cerebral cortex to neuromuscular junction and muscle either damaging one area (e.g., Purkinje cell, presynaptic cholinergic synapses) or multiple areas (e.g., encephalomyelitis) which could present with more global deficits. |
|  | Hematologic Malignancy | Hyperleukocytosis/leukostasis - neurological signs and symptoms include visual changes, headache, dizziness, tinnitus, gait instability, confusion, and somnolence. |
| **Endocrine/**  **Nutrition** | Dehydration | Older adults are prone to dehydration due to impaired thirst mechanisms and an inability to increase water intake due to compromised mobility and/or swallowing ability. Presentation of dehydration in older adults is often nonspecific. Dehydration may lead to electrolyte abnormalities (hyper or hyponatremia) or renal failure, which could cause confusion. |
|  | Hypothyroidism | More likely to present with chronic picture of mild cognitive impairment, as opposed to acute confusion |
|  | Hyperthyroidism | Hyperthyroidism in older patients may present with apathy, rather than hyperactivity, tremor, and other symptoms of sympathetic overactivity. |
|  | Hypoglycemia | Would ask about history of diabetes, liver disease, or prolonged fasting |
|  | B12 deficiency | Classic findings associated with vitamin B12 deficiency include severe macrocytic anemia, jaundice, and variable neurologic abnormalities, including mental sluggishness, shuffling gait. |
|  | Hypercalcemia | Neurologic symptoms (lethargy, confusion, stupor, and coma) may occur in patients with severe hypercalcemia (>14 mg/dL) |
|  | Thiamine deficiency (Wernicke encephalopathy) | Classic triad: Encephalopathy, Oculomotor dysfunction, Gait ataxia  Associated with chronic alcohol use, can also result from very low nutritional intake for other reasons |
|  | See iatrogenic below for medication toxicity |  |
| **Toxic/Metabolic** | Alcohol or benzodiazepine withdrawal | Can present with acute confusion. May also see hand tremor, tongue fasciculation, hallucinations, tachycardia, and/or hypertension |
|  | Hyponatremia | Multiple etiologies, in this case most likely due to SIADH from SSRI. |
| **Neurologic** | Ischemic or Hemorrhagic stroke | Patient with history of CVA and atrial fibrillation, important to ask about adherence with anticoagulation medication |
|  | Subdural Hematoma | Subdural hematoma can present with confusion. It is important to ask about falls. |
|  | Normal Pressure Hydrocephalus | NPH is associated with a classic triad of dementia, gait disturbance, and urinary incontinence |
|  | Seizure/post-ictal vs non-convulsive status epilepticus | May have history of seizure followed by confusion or ongoing confusion in the case of non-convulsive status epilepticus. |
|  | Pain (from skin breakdown or infection, UTI, unknown trauma) | Undertreated pain and inadequate analgesia may be risk factors for delirium in frail older adults. |
|  | “Sundowning” | Delirium should be distinguished from "sundowning” phenomenon of behavioral deterioration seen in the evening hours, typically in patients with dementia. |
| **Psychiatric** | Change in Caretaker/Surroundings | Contributing as above |
|  | Sensory Deprivation vs Overstimulation | Contributing as above |
| **Infectious Disease** | Upper airway Infection/Aspiration PNA | Patients with dementia are at risk for aspiration, which may cause pneumonia or pneumonitis. Older adults with pneumonia may present with confusion as one symptom. |
|  | Urinary Tract Infection | UTIs are common in older adults and may present in subtle fashion in this group with change in mental status as one possible symptom along with urinary symptoms and systemic signs of infection (fever, chills, etc.) |
|  | Infected pressure ulcer | Pressure ulcers -- areas of tissue necrosis that tend to develop when soft tissue is compressed between a bony prominence and an external surface for a prolonged period. Skin breakdown can predispose older adult to cellulitis, osteomyelitis, or bacteremia |
|  | Encephalitis/CNS Infection | Travel history, recent exposure to mosquitos/bug bites would be important components of history |
|  | Prosthetic Device infections | The prevalence of implanted prosthetics increases with age (prosthetic joints, valves, pacemakers, vascular grafts). These foreign bodies may serve as nidus of infection. |
|  | Bacteremia and Sepsis | Older patients with bacteremia are less likely to have fever or chills than younger patients. |
|  | Endocarditis | More common in patients with degenerative valvular disease, MVP, or prosthetic valve. |
| **Iatrogenic** | Medication classes known to predispose to delirium | Anticholinergics, steroids, antiarrhythmics including digoxin, antidepressants, anxiolytics, diuretics, fluoroquinolones |
|  | Polypharmacy | # of meds used to define "polypharmacy" is variable, but generally ranges from 5 -10  Increased risk of adverse reaction to meds with age, due to metabolic changes and decreased drug clearance associated with aging, this risk is compounded by increasing numbers of drugs used. |

1. **Problem Representation:** When the students have completed gathering the history, they will likely have narrowed down their hypotheses to 3-4 most likely and/or “do not miss” diagnoses. Have the students work as a group to write a problem representation on the white board. The problem statement should be an accurate representation of the problem using semantic qualifiers that will lead to the most productive hypotheses and illness scripts. The problem representation should include: *Who is the patient? What is the context? What is the problem?*

**Example: MJ is a 97-year-old woman with moderately severe cognitive decline, atrial fibrillation with history of a stroke managed on apixaban, hypertension, and chronic constipation, recently started on SSRI presenting with acute onset of confusion.**

Potential Questions for Discussion if time allows: *Why did we choose to include a history of cognitive decline, atrial fibrillation, recent SSRI initiation, and constipation as part of the context? Are there other aspects of context that could be included as well?*

**Hypothesis-Driven Physical Diagnosis (10 minutes):**

Students will divide into groups of 2-3 and write on the board (1) which aspects of the physical exam they would perform in this patient and (2) how specific findings in these body systems will help to confirm or refute their remaining diagnostic hypotheses. Table 2 includes some examples of what students may generate. After about 5 minutes, have the groups re-convene and compare what they have each discussed. After the groups report out, the faculty facilitator will review each of the slides related to the physical exam in Appendix B and students can compare their hypotheses with the patient’s exam.

| **Table 2. Potential Hypothesis-Driven Physical Examination and findings for this case** | |
| --- | --- |
| **General:** | - - - - - Pallor or Jaundice might be suggestive of anemia or liver dysfunction.         - Bitemporal wasting may suggest malnutrition         - Skin turgor, though a traditional method of assessing hydration, lacks precision. When used in the elderly, turgor is best tested on the inner aspect of the thigh or over the sternum |
| **Vitals:** | - - - - - Temperature – may see hyper or hypothermia in infection         - HR – tachycardia may be seen in volume depletion, infection, arrythmia         - BP – evaluate for hyper or hypotension; assess for orthostasis |
| **HEENT:** | - - - - - Conjunctiva – may have pallor in anemia         - Sclera – icterus in liver failure         - Mucous membranes – dry in volume depletion         - Dentition – missing teeth, ill-fitting dentures may impact ability to eat regular diet |
| **Neck:** | - - - - - Multinodular goiter – may indicate thyroid disease |
| **Pulmonary:** | May be challenging to position patient in a manner to allow effective evaluation of posterior back. May need to roll patient onto her side in the bed or ask daughter for assistance in sitting patient forward to allow auscultation, ability to percuss will be very limited in that position.  Aspiration PNA or pneumonitis more likely to effect RLL and RML due to the larger caliber and more vertical orientation of the right mainstem bronchus, may hear coarse crackles and/or focal wheezes  CHF - Bibasilar crackles |
| **CV:** | - - - - - Jugular venous distention – may be seen with decompensated heart failure         - Irregular heart rhythm – may indicate atrial fibrillation         - S3 – may be seen with decompensated heart failure         - Leg edema – may indicate acute decompensated heart failure, renal failure, decompensated cirrhosis with portal hypertension, or chronic venous insufficiency         - Sacral edema – may be where edema is apparent in patients who have been supine for extended periods |
| **Abdominal:** | - - - - - Suprapubic tenderness/distention – urinary retention         - Abdominal mass/firmness - constipation |
| **Rectal:** | - - - - - Fecal Impaction: impacted stool may be hard, but key to the diagnosis of fecal impaction is in finding a copious amount of stool in the rectum. |
| **Skin:** | - - - - - Skin breakdown/Infection – assess all points of increased pressure for signs of skin breakdown/ulcer formation. Full skin assessment requires uncovering the body. Particular attention should be paid to sacrum, hips, and heels |
| **Neurologic:** | - - - - - Level of consciousness – important to assess for delirium and dementia         - Attentiveness – inattention in delirium         - Differentiate Delirium from Dementia - Assess level of consciousness         - Consider how you to respectfully pose orientation questions to an older adult         - Consider how you will perform a neurologic exam if a patient is not consistently following commands |

**Group Discussion (10 minutes):**

Bring students back together around the table and share abnormal findings from the PowerPoint (Appendix B). Students should be encouraged to consider how the physical examination findings differential would relate to the different findings and/or how this information might help discriminate amongst diagnoses.

Potential teaching points for Appendix B

- **Slide 2:** This slide shows the patient from the case’s vital signs and general appearance. Notable findings are normal vital signs aside from lower blood pressure. We do not know her baseline blood pressure, but we do know that she has a diagnosis of hypertension and takes hydrochlorothiazide, so this may be relative hypotension. This could be due to volume depletion. The general section suggests inattentiveness and increased somnolence.
- **Slide 3:** Skin turgor, though a traditional method of assessing hydration, lacks precision. When used in the elderly, turgor is best tested on the inner aspect of the thigh or over the sternum.
- **Slide 4:** Arcus senilis is a white or gray opaque ring in the corneal margin which can present at birth but more commonly appears in those older than 50 and resulting from cholesterol deposits. Bitemporal wasting is suggestive of more longstanding muscle wasting. This patient does not have teeth, which may have an impact on her oral intake depending on dentures etc. Mildly dry mucous membranes, which suggests hypovolemia.
- **Slide 5:** The irregularly irregular rhythm suggests that she is currently in atrial fibrillation. Her pulmonary exam is normal. Her mobility assessment gives essential information about the patient’s functional status.
- **Slide 6:** The skin exam shows no skin tenting over the sternum, although this does not rule out volume depletion. The skin exam looks for pressure injuries and describes what is likely a stage 1 pressure injury (intact skin but with non-blanching redness for >1 hour after relief of pressure)
- **Slide 7:** Abdominal and rectal exams without suggestion of constipation or urinary retention. Neurologic exam with facial droop and right sided weakness, which are sequelae of her previous stroke. Note that static neurologic deficits can worsen in setting of metabolic derangements in the absence of new neurologic insult/injury. She is disoriented, inattentive, and sleepy.
- **Slide 8: Ask** students what they would want to do next prior to proceeding to the next slide.
- **Slide 9:** This list of options includes hyperlinks for additional information about each treatment option. If you click on any of the links, you can click on the “work up” link in the lower right-hand corner to return to slide 8.
- **Slide 10:** MOLST (Medical Orders for Life-Sustaining Treatment; note: in some states this is referred to as a POLST (Physician Orders for Life-Sustaining Treatment form) form completed in 2022 clearly documents the patient’s wish to avoid hospitalization. This form documents that the patient is DNR, would not want to be transported to the hospital, and would want treatment for any reversible medical conditions that could be accomplished in the home. Note that hospitalization itself is a cause of delirium.
- **Slide 11:** Glucose is normal.
- **Slide 12:** Metabolic panel shows low serum sodium (hyponatremia), which may be trigger for her delirium. There are many possible etiologies of hyponatremia. In this case the most likely possibilities would be SIADH due to recently started SSRI or hypovolemic hyponatremia due to recent decrease in oral intake.
- **Slide 13:** Recent TSH was checked and normal.
- **Slide 14:** An EKG could be done in the home. But would ask the students specifically what information they would be looking for if an EKG is obtained. She has a known history of atrial fibrillation, and her arrhythmia was heard on the physical exam, and so seeing this on an EKG would not add additional information.
- **Slide 15:** A chest x-ray could be done in the home. But would ask the students specifically what information they would be looking for if a chest x-ray is obtained. She does not have any respiratory symptoms or underlying lung disease, and her pulmonary exam was unremarkable, so the index of suspicion for a pulmonary diagnosis is low.
- **Slide 16:** Her hemoglobin is low. It is common to see anemia in older adults. If within goals of care may merit an investigation into the etiology, but it is not likely to be the reason for her confusion. No leukocytosis is another piece of data arguing against infection such as urinary tract infection or pneumonia
- **Slide 17:** Her urinalysis does not have any pyuria or nitrites, which makes a urinary tract infection unlikely
- **Slide 18:** If blood cultures are drawn note that they won’t have a preliminary result for 24 hours and will not be finalized for 72 hours.
- **Slide 19:** Ask the students, “*Given the subjective and objective information we have gathered for this patient what would be your Summary Statement (*revised problem representation – who is the patient, what is the context, what is the problem, inclusive of pertinent PE and lab findings)?” You may choose to have the student do a pair and share or work together as a large group to develop the summary statement.

Sample Summary Statement:

*MJ is a 97-year-old woman with moderately severe cognitive decline, atrial fibrillation complicated by a stroke managed on apixaban, recently started on SSRI presenting with acute onset of confusion and found to have new hyponatremia likely secondary to medication induced SIADH.*

- **Slide 20:** This is the checklist for students to use as a guide when practicing the oral presentation. See below for further instructions.

**Oral Presentation Practice (20 minutes):**

Keep slide 20 open for students to reference during the oral presentation. Allow students a few minutes to arrange their thoughts in preparation for presenting the chief concern and HPI to the group. Then ask for a volunteer to present. Break the group into thirds and ask each to listen for one section of the oral presentation checklist (opening statement, HPI or presentation style). Following the presentation, ask the presenter: *How did that go? What did you do effectively?* Ask the observers to share specific feedback with the presenter on their assigned section. As time allows, repeat to give additional students the opportunity to present.

**Wrap-up (5 minutes):**

Faculty should ask each student to identify their take-home point and share it with the group -- something they learned from today’s session that they plan to continue to practice.

**Appendix: Patient Case Notes for Role Play**

**Patient name:** Mabel Johnson

**Date of Birth:** 2/14/19*XX* (*year to be updated as needed to reflect patient age of 97*)

**Setting: House Call Visit**

**Source:** Patient’s daughter Jane Reynolds (or son Richard Johnson)

**CC:** “I am so glad you were able to come out today, my mom has not been herself since last night.”

**HPI:**  Mabel Johnson is a 97-year-old woman with a past medical history of moderately severe dementia, mild hypertension controlled on HCTZ, atrial fibrillation complicated by a stroke 2 years ago with mild residual right facial droop and right upper extremity weakness managed on apixaban, and glaucoma who lives at home with her daughter Jane now with acute onset in change in mental status for the past 1-2 days.

**Patient’s baseline:** Mabel was recently enrolled as a new patient in the house calls program, which provides access to physician home visits.  Jane describes that at baseline Mabel is dependent for many activities of daily living.  They have a private pay home health aide who comes into the house 10-12 hours per day 6 days per week depending on Jane’s work schedule.  Jane and the home health aide (HHA) get Mabel out of bed every morning and walk with a walker to a recliner chair in her bedroom and a short distance down the hallway where she takes her meals. She has not had any falls.  She spends most of the day in the recliner watching television, listening to music, and singing or paging through magazines.  The bathroom off her bedroom has been fully equipped with ADA compliant doorways, shower chair and grab bar which allows Jane and the HHA to assist Mabel with bathing 3 times per week.  Mabel speaks in single words or short sentences and seems to recognize her close family members as she will occasionally use their names, but always greets them with a giant smile.  She is incontinent of urine, using adult diapers throughout the day and night.  She has a hospital bed in her room which they have found helpful in sitting her up and getting her cleaned and ready each day.

**Current Problem:** She sleeps well but for the past 2 nights she has been waking up a few times per night and singing for about 30-60 minutes before falling back to sleep.  Mabel generally has a good appetite; she eats a full, unrestricted diet of food prepared by family.  Last night at dinner, Mabel could make her way down the hall with her walker with more difficulty than normal, pausing every few steps.  She did not eat much and when Jane tried to coax her to eat, she shut her mouth tightly.  Mabel did not express any discomfort of any kind and when Jane asked her if she felt any pain or nausea, she said no.

This morning Jane noted that her mom was slumped to the right in the bed and required calling her name several times before she opened her eyes, her face looked like “the right side is droopier than usual” and she needed assistance just to reposition herself in the bed.  She offered her mom some OJ thinking she “might have low sugar after not eating dinner” but Mabel started coughing with her 1^st^ few sips and then shut her mouth tightly.

She has no personal or family history of diabetes nor has she had any history of hypoglycemic episodes in the past. Jane is not aware of any fever, chills, or sick contacts.  She has not had any change in weight recently or reports of her heart racing.  Jane has not noted any change in the frequency of wet diapers nor in odor or color of urine.  Mabel has a lifelong history of constipation, particularly worsened after her decreased mobility after her stroke 2 years ago. She has a bowel movement every 2-3 days, managed by drinking prune juice every day with breakfast and adding stewed prunes if she hasn’t had a BM by the 3^rd^ day, her last BM was probably “5 maybe 6 days ago”.

Jane was away for her niece’s wedding in Virginia this past weekend and she privately hired someone to spend the weekend with her mom from “a very reputable agency.”  The aide seemed to have it together, and left her with detailed notes, but Jane felt that her mother was not cared for as well as she would have liked. Jane noted some redness on her mother’s backside, so she questions the aide’s report that her mother was out of bed to the kitchen and her recliner on her usual schedule during the time away.  Jane feels very guilty about having left her mother, she had been debating the trip for months, but friends and family encouraged her to go as she has not been on a vacation since her mother came home from rehab after her stroke 2 years ago.

At the time of enrollment in the house calls program, 3 weeks ago, she was started on a low dose of sertraline as this was something her PCP and Jane had been considering for a while.  Jane had been concerned that her mother’s mood had seemed down for quite some time as she had lost interest in leaving the house, used to enjoy getting outside on the backyard patio or driving around the neighborhood or up by the water in Little Neck.  Jane started giving her mom the sertraline 25mg about 2 weeks ago hoping it would prevent her mood from worsening while she was away.

Mabel has clearly expressed to her family in the past (and Jane is supportive) that she never wants to go back to the ED, hospital, or rehab/nursing home as she had a prolonged and unpleasant stay after her stroke 2 years ago.

**Timeline Summary:**

- **3 weeks ago: started** on sertraline.
- **6 days ago:** daughter traveled, and the patient was left with a new 24h private pay home health aide.
- **2 nights ago:** started waking up at night singing for 30-60 minutes.
- **Last night:** trouble getting down hallway to dinner table, did not eat much.
- **This morning:** sleepy, slumped to right side, more right facial droop, coughing after drinking a few sips of orange juice.

**Review of Systems:** limited due to patient condition

General: Daughter reports patient has been losing weight slowly over the last 2 years, as above

HEENT: Patient wears reading glasses x 30 years, no visual concerns reported.

Pulmonary: No shortness of breath, cough as above with drinking juice this morning

Cardiovascular: No chest pain

Abdomen: as above, no vomiting

Neurologic: as above

Musculoskeletal: as above

**Past Surgical History:** Several dental extractions over the past 20 years for caries, no complications, has not required any dental procedures since the starting of her anticoagulation 2 years ago.

**Past Medical History:**

Moderately severe cognitive impairment x 3-4 years

Mild hypertension, controlled x 30 years

Atrial fibrillation s/p CVA 2 years ago with mild residual right facial droop and right upper extremity weakness managed on apixaban.

Glaucoma x 10 years

**GYN History:** Pt had 4 NSVD, post-menopausal x 50 years, no history of sexually transmitted infections, not sexually active x at least 15 years when husband died, husband was her only partner (to best of daughter’s knowledge)

**Medications:**

Donepezil 10 mg PO daily

Sertraline 20 mg PO nightly

Apixaban 5mg PO twice daily

Hydrochlorothiazide 12.5 mg PO daily

Multivitamin 1 tablet PO daily

Polyethylene Glycol 17 g PO daily

Cholecalciferol 5000IU 1 tablet PO daily

Calcium 500mg gummy vitamin PO twice daily

Timolol 1 drop to each eye nightly

Latanoprost, 1 drop to each eye nightly

Omeprazole 40 mg PO daily (started when she was in the hospital after her CVA 2 years ago)

**Allergies:** no known drug, food, environmental allergies

**Immunizations:** had flu, COVID, and RSV vaccines in the fall, pneumovax and shingles vaccine last administered 5 years ago when patient 1^st^ transferred care to geriatrics practice from PCP who retired

**Social History:** grew up in South Carolina but moved to NYC for work after WWII, married husband and settled in Queens where they raised their 4 children. She currently lives with daughter Jane in a 1 story home in Bayside, Queens. She has access to healthy food.  She completed high school and then began working as a secretary in a bank in Manhattan until birth of her 1^st^ child, was homemaker and then went back to work as school secretary in local public school when youngest child started kindergarten.  She retired from that job 22 years ago at the age of 75.  Lived independently up until her stroke  2 years ago, but family had noticed prior to that for 12-18 months that she was not quite herself – her home which used to be pristine was starting to get dusty and cluttered, patient used to make elaborate meals and desserts for family gatherings but she started to forget her recipes or add or subtract ingredients and had less attention to her presentation style.  Jane and her siblings were just discussing hiring help for Mabel who had been resistant vs moving her in with one of them when she had her CVA. The family pays the HHA privately. She has Medicare insurance with supplemental insurance.

**Habits:** Patient never smoked tobacco, used to enjoy drinking beer or wine at family gatherings but not regularly, will still have some on occasion for celebrations.

**Family History:** Youngest of 6 siblings, she is only surviving, next oldest sister died of “old age” at 98 about 2.5 years ago which seemed to contribute to patient’s decline.  2 brothers died in WWII, 2 other sisters died in their early 90s, one with Alzheimer’s, the other had CHF. 4 children aged 75, 73, 72 and 68 (Jane) are all in good health.
