# Supplementary material for: Integration of Geriatrics and Palliative Medicine Into a Medical Student Clinical Reasoning Curriculum
Source: MedEdPORTAL. 2025 Feb 6;21:11495. doi: 10.15766/mep_2374-8265.11495 (PMC11799358; doi:10.15766/mep_2374-8265.11495)
Supplement: Supplementary file 1 — Facilitator Guide.docxPhysical Exam Findings.pptxStudent Survey.docxFaculty Survey.docx [file mep_2374-8265.11495-s001.zip › B. Physical Exam Findings.pptx]

## Slide 1
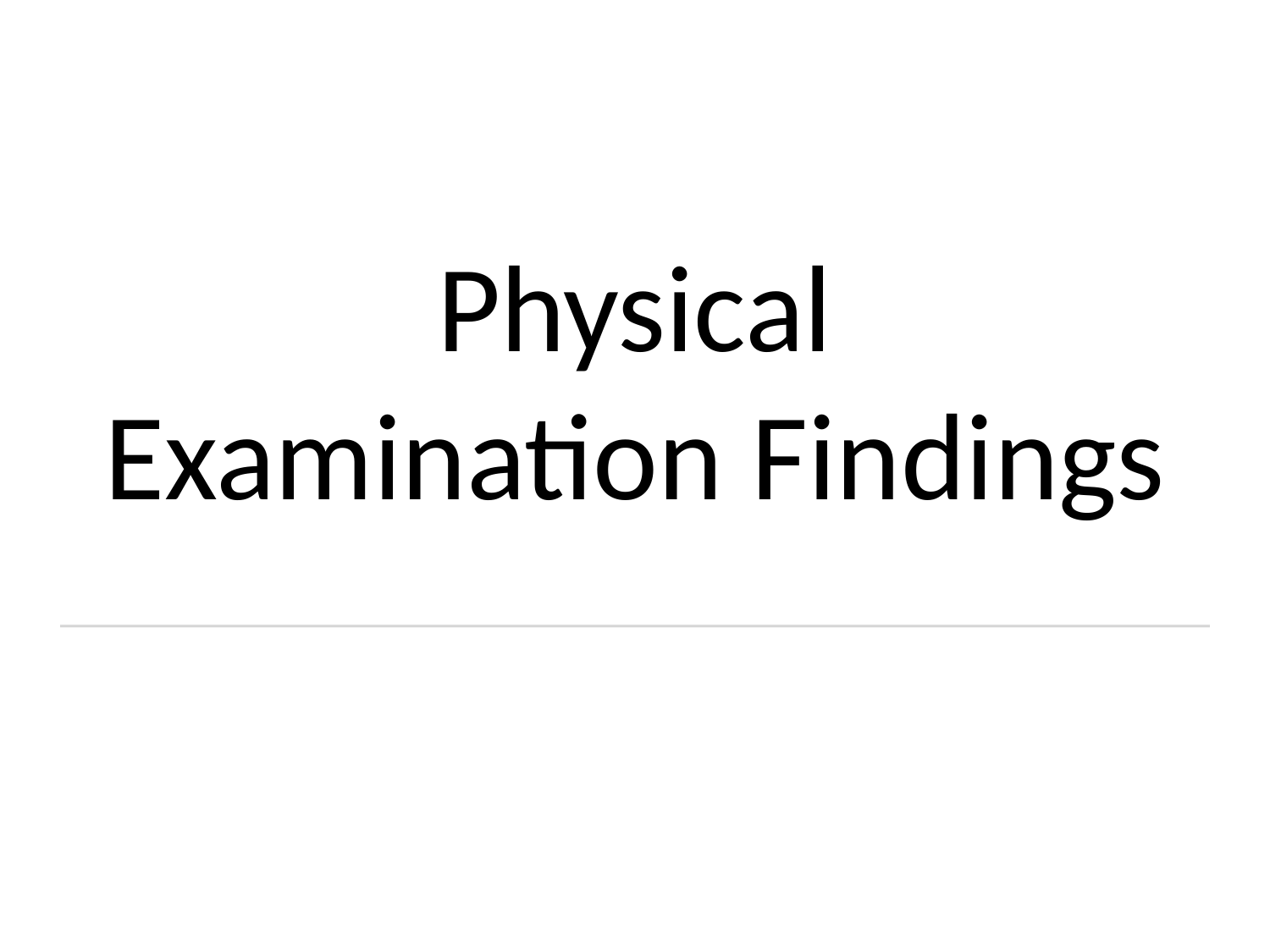

# Physical Examination Findings

## Slide 2
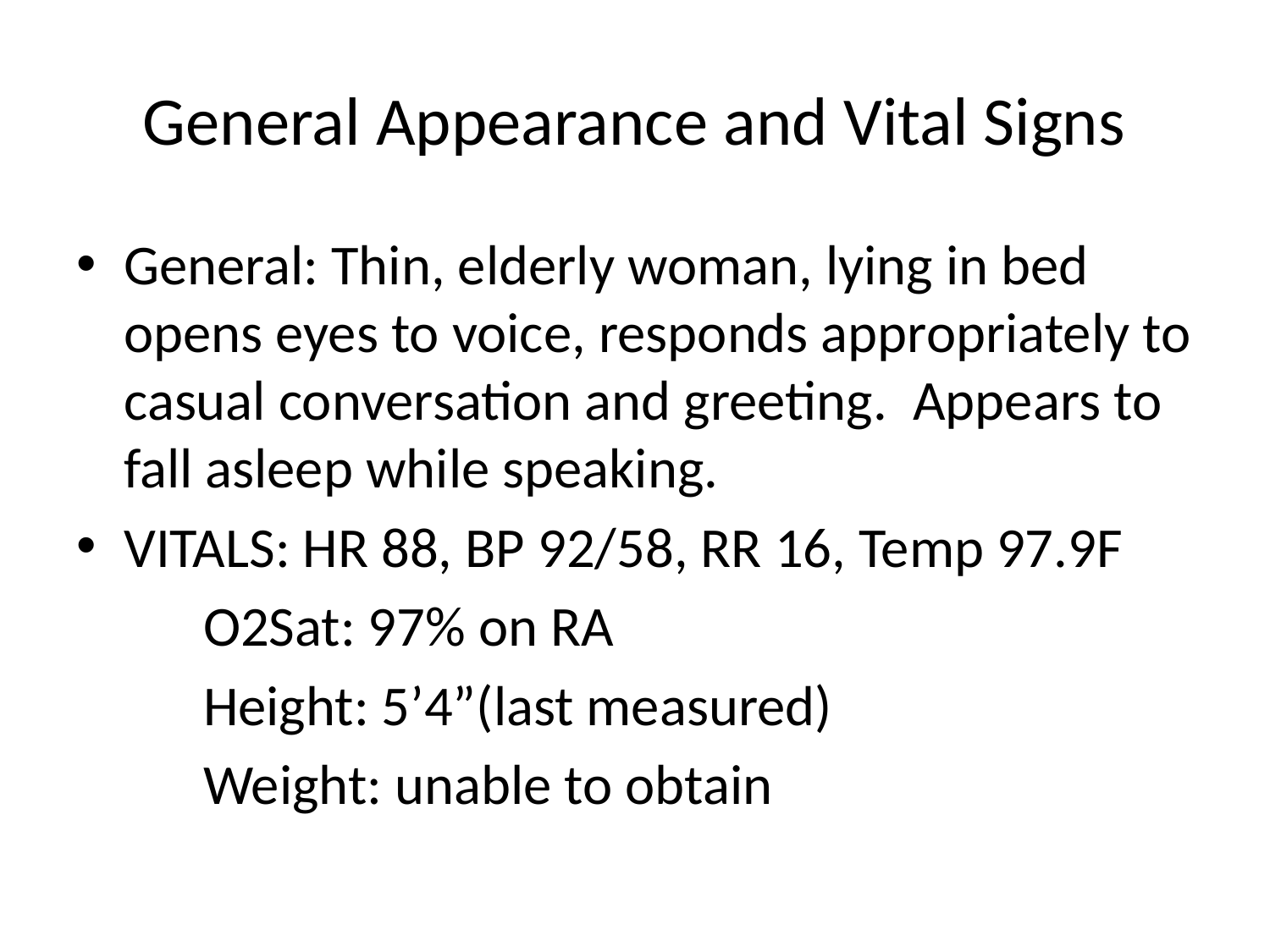

# General Appearance and Vital Signs
General: Thin, elderly woman, lying in bed opens eyes to voice, responds appropriately to casual conversation and greeting.  Appears to fall asleep while speaking.
VITALS: HR 88, BP 92/58, RR 16, Temp 97.9F
	O2Sat: 97% on RA
	Height: 5’4”(last measured)
	Weight: unable to obtain

## Slide 3
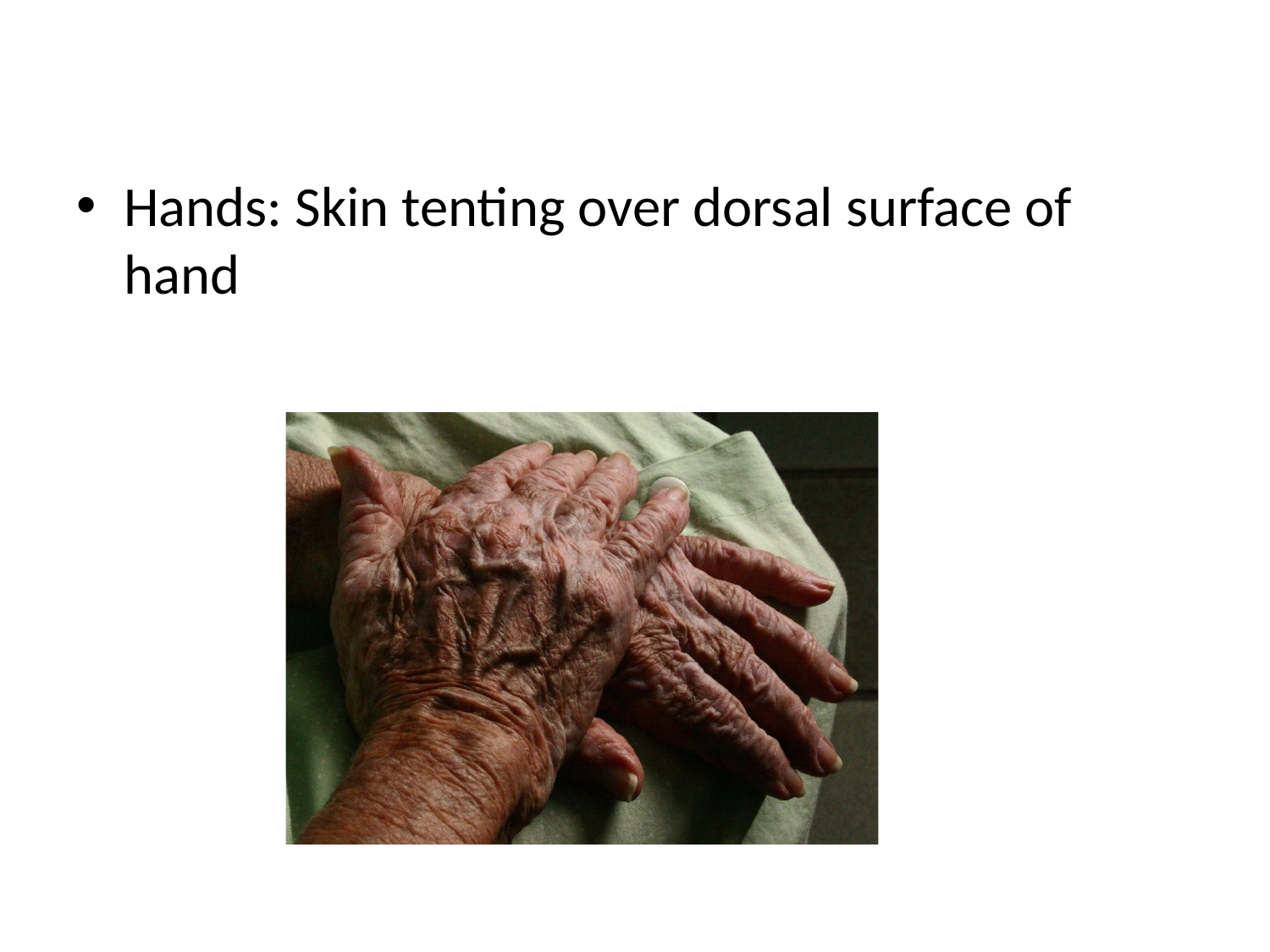

Hands: Skin tenting over dorsal surface of hand

## Slide 4
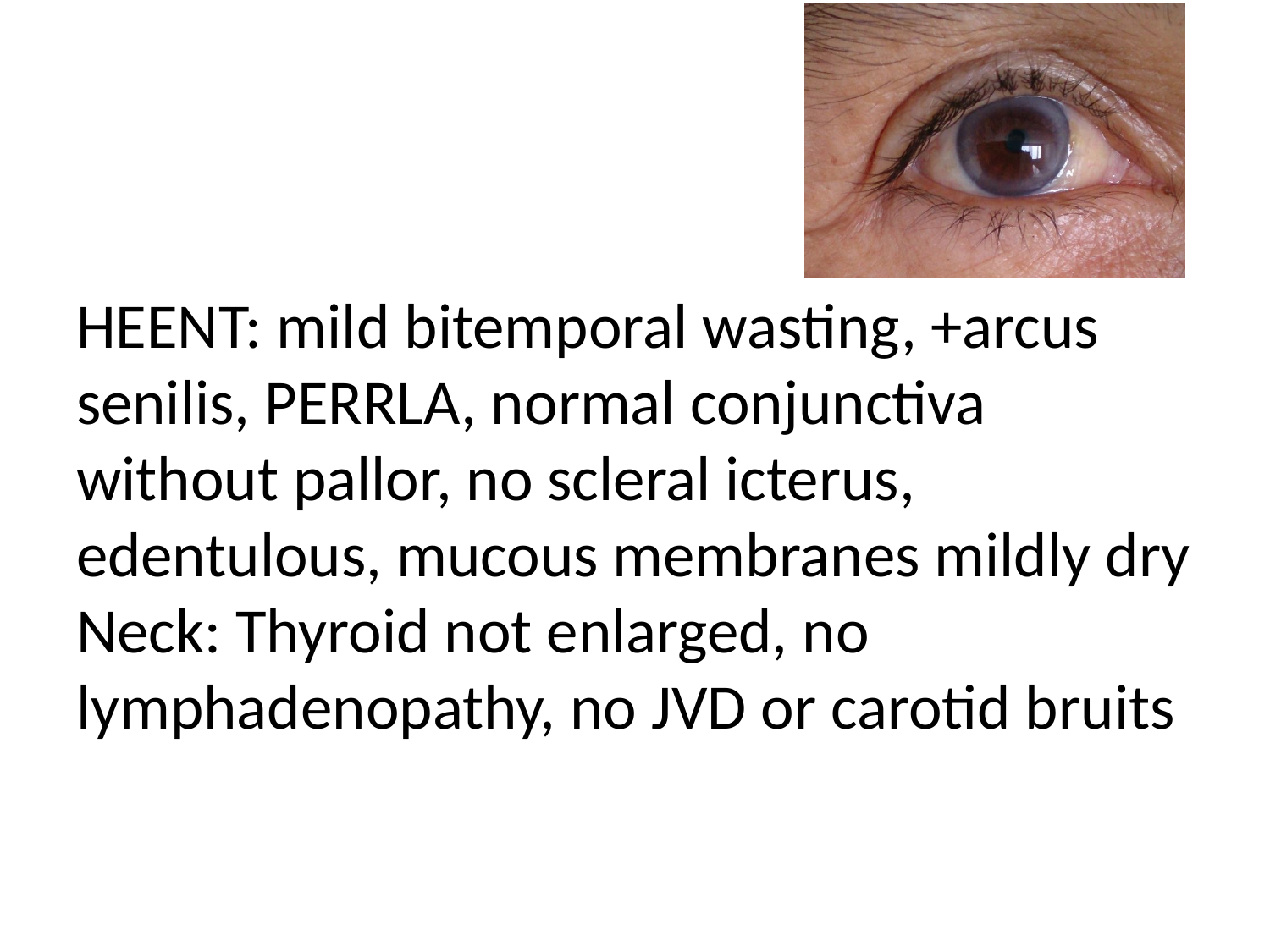

# HEENT: mild bitemporal wasting, +arcus senilis, PERRLA, normal conjunctiva without pallor, no scleral icterus, edentulous, mucous membranes mildly dry Neck: Thyroid not enlarged, no lymphadenopathy, no JVD or carotid bruits

## Slide 5
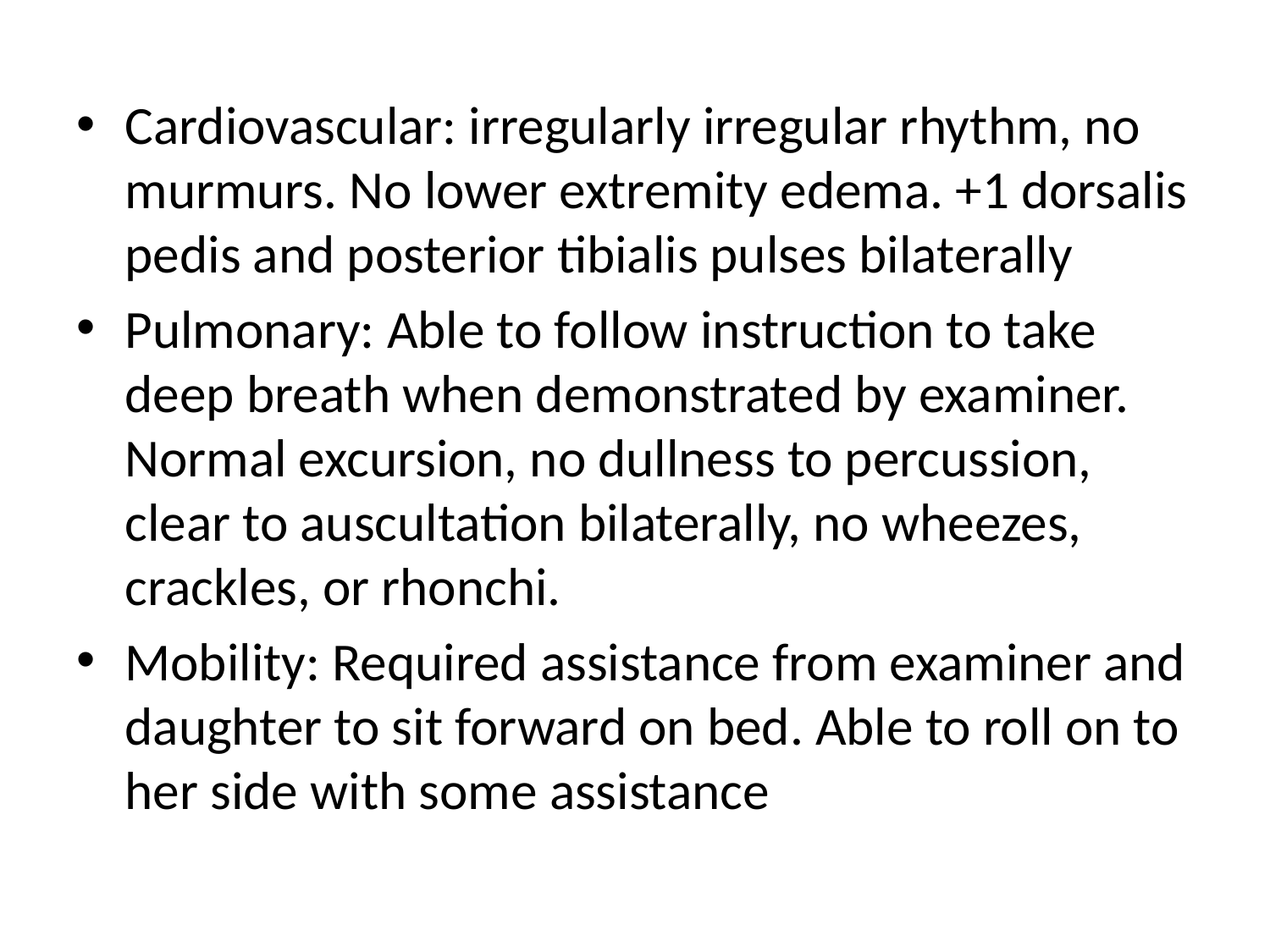

Cardiovascular: irregularly irregular rhythm, no murmurs. No lower extremity edema. +1 dorsalis pedis and posterior tibialis pulses bilaterally
Pulmonary: Able to follow instruction to take deep breath when demonstrated by examiner. Normal excursion, no dullness to percussion, clear to auscultation bilaterally, no wheezes, crackles, or rhonchi.
Mobility: Required assistance from examiner and daughter to sit forward on bed. Able to roll on to her side with some assistance

## Slide 6
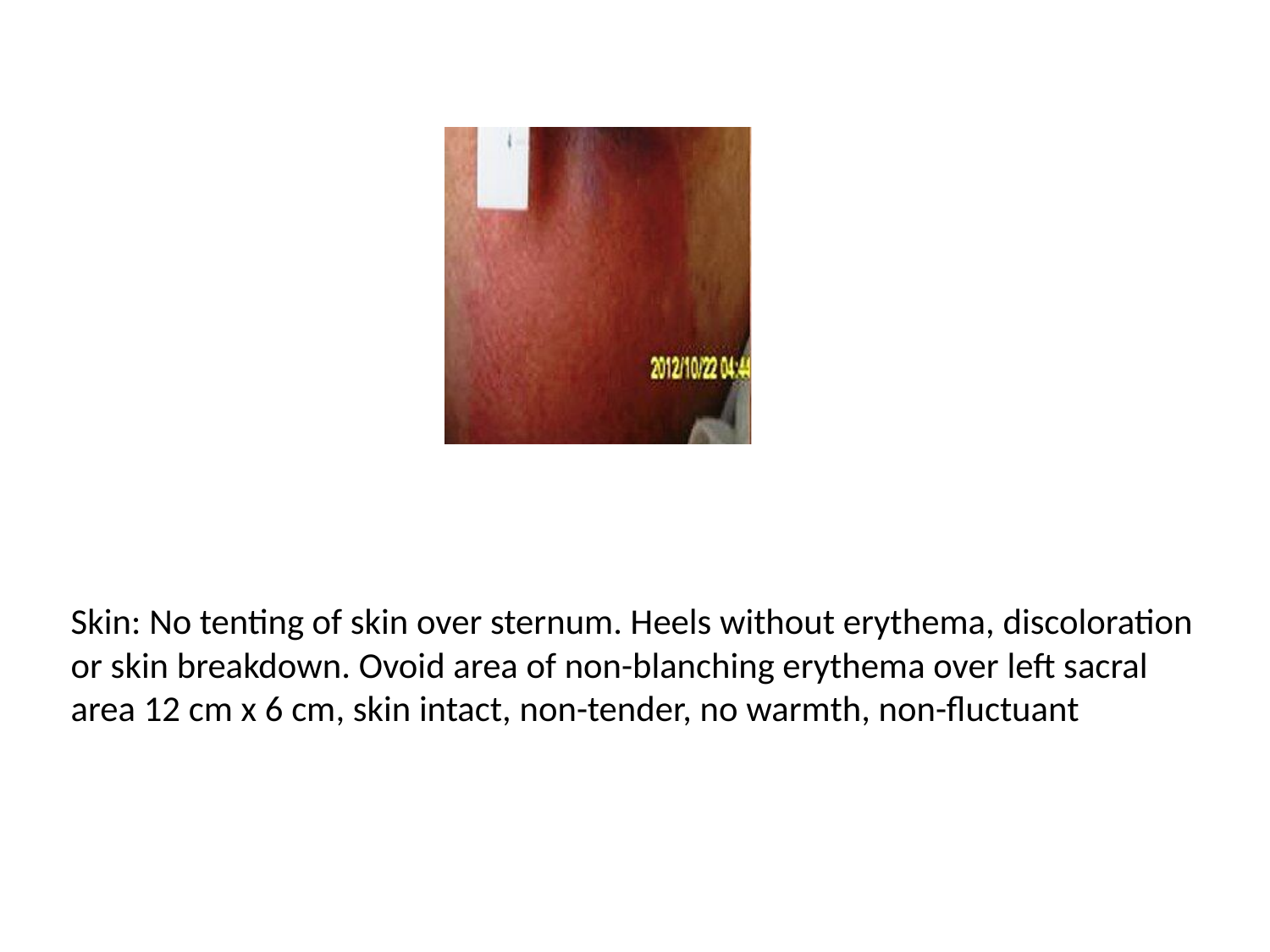

Skin: No tenting of skin over sternum. Heels without erythema, discoloration or skin breakdown. Ovoid area of non-blanching erythema over left sacral area 12 cm x 6 cm, skin intact, non-tender, no warmth, non-fluctuant

## Slide 7
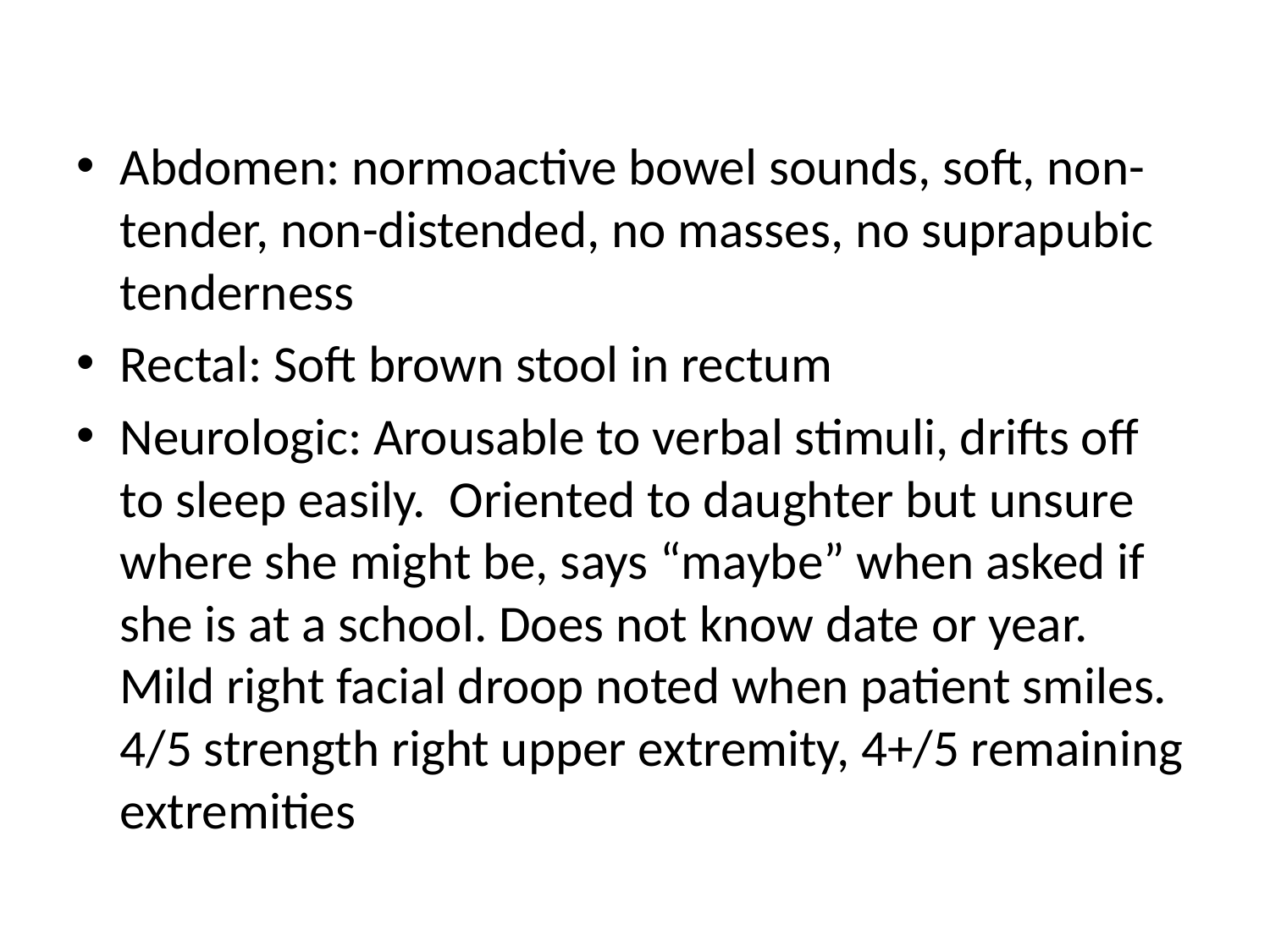

Abdomen: normoactive bowel sounds, soft, non-tender, non-distended, no masses, no suprapubic tenderness
Rectal: Soft brown stool in rectum
Neurologic: Arousable to verbal stimuli, drifts off to sleep easily. Oriented to daughter but unsure where she might be, says “maybe” when asked if she is at a school. Does not know date or year. Mild right facial droop noted when patient smiles. 4/5 strength right upper extremity, 4+/5 remaining extremities

## Slide 8
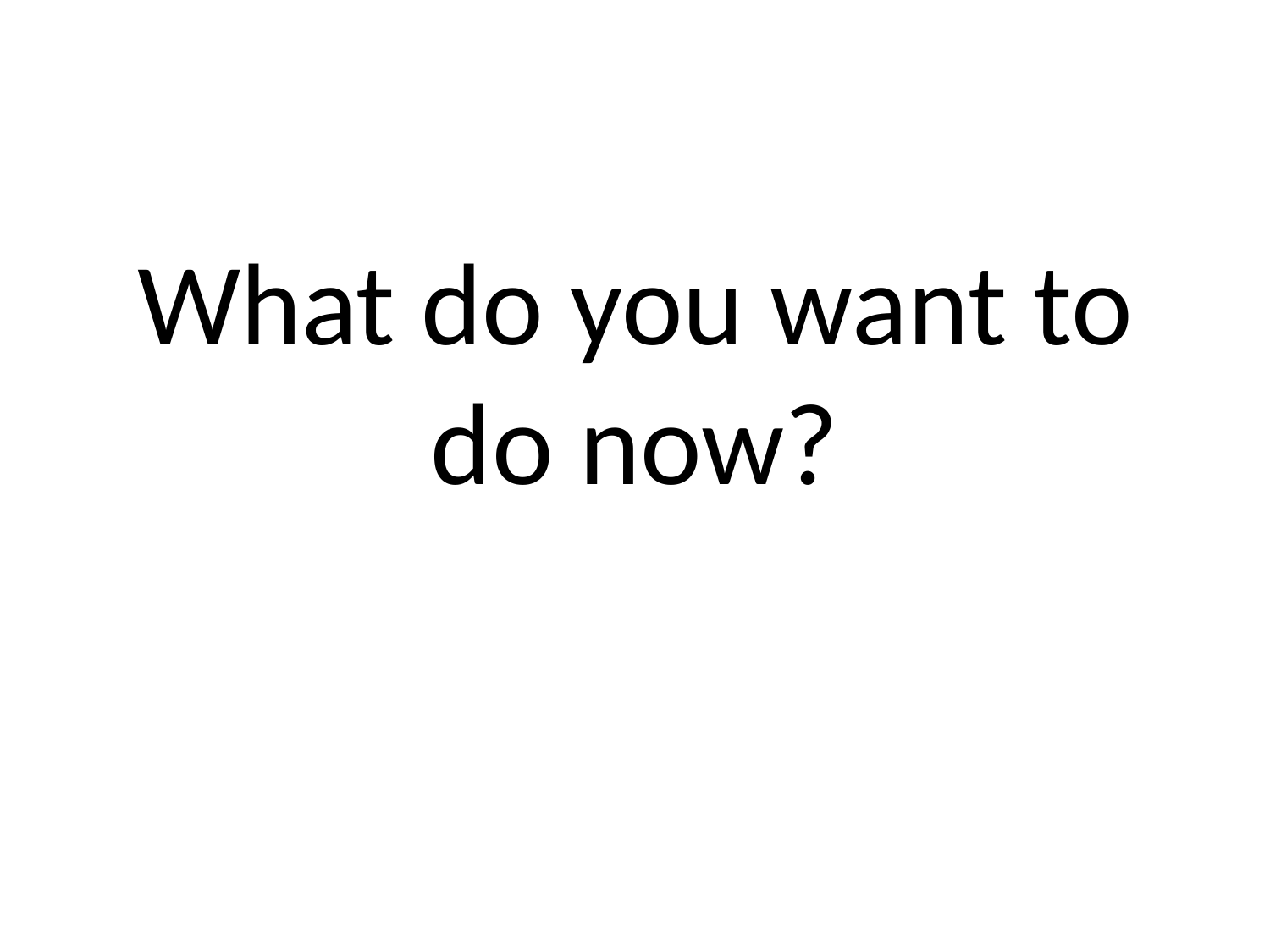

#
What do you want to do now?

## Slide 9
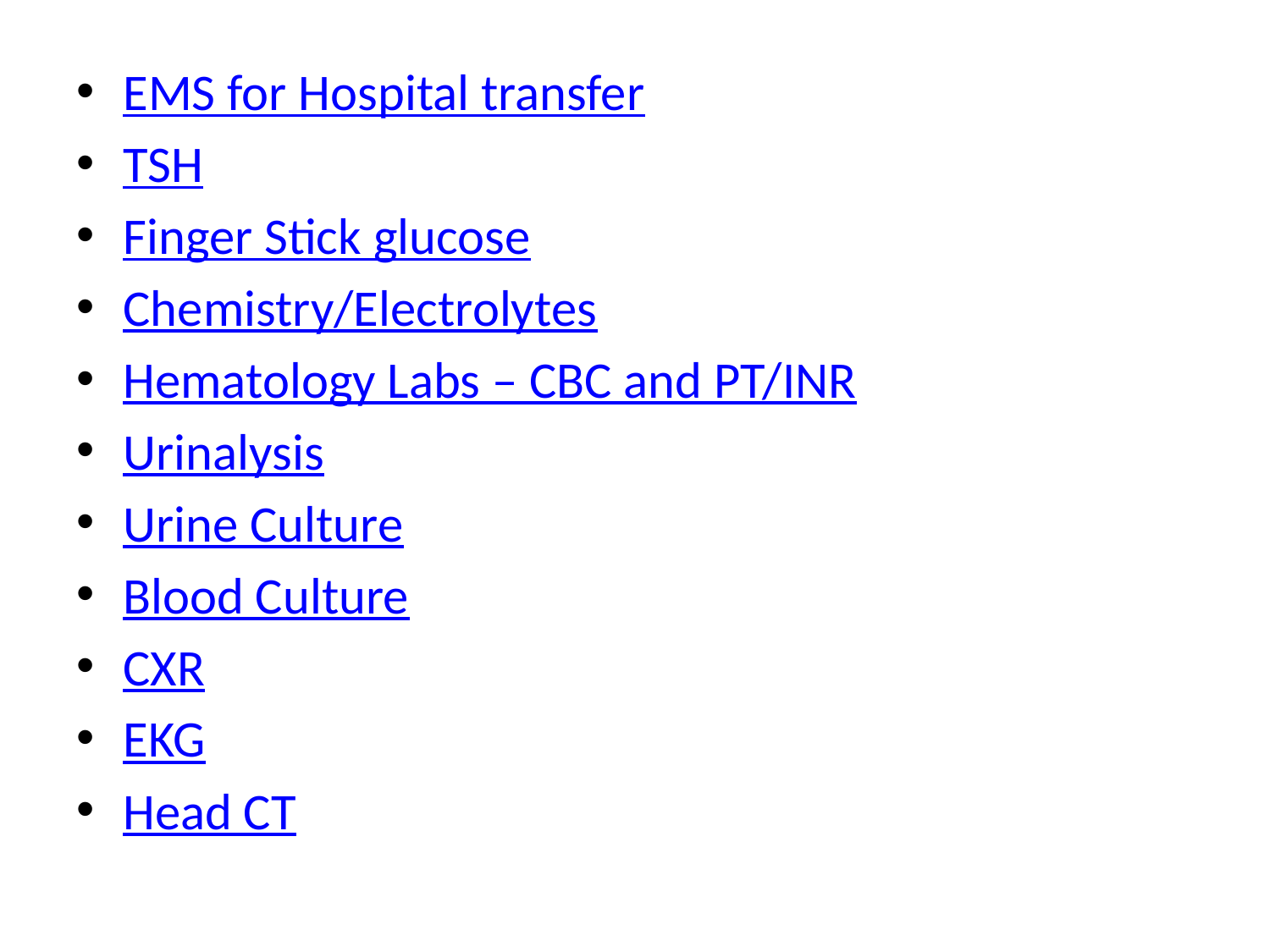

EMS for Hospital transfer
TSH
Finger Stick glucose
Chemistry/Electrolytes
Hematology Labs – CBC and PT/INR
Urinalysis
Urine Culture
Blood Culture
CXR
EKG
Head CT

## Slide 10
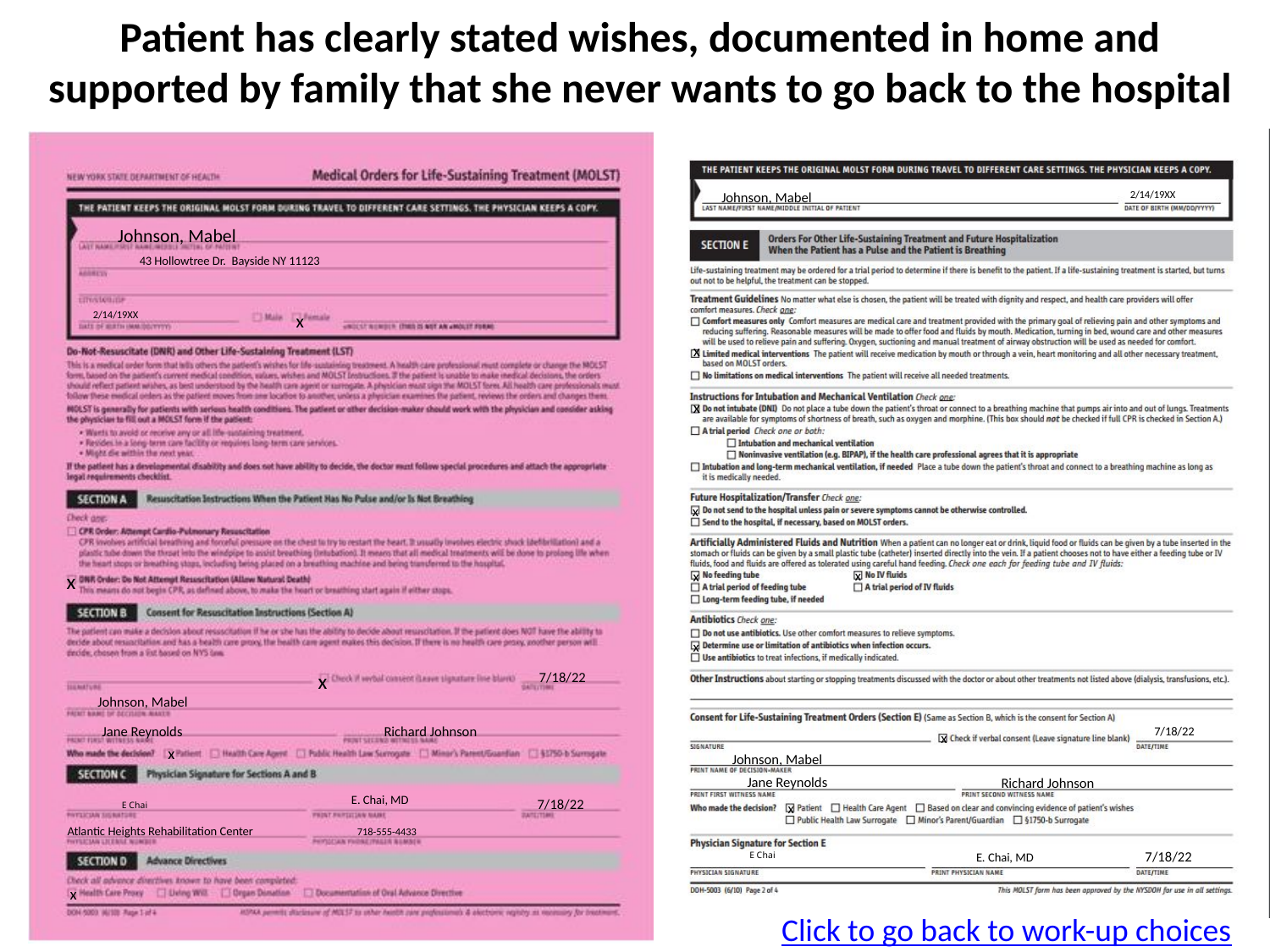

# Patient has clearly stated wishes, documented in home and supported by family that she never wants to go back to the hospital
2/14/19XX
Johnson, Mabel
Johnson, Mabel
43 Hollowtree Dr. Bayside NY 11123
2/14/19XX
x
x
x
x
 x
x
x
x
x
7/18/22
Johnson, Mabel
Jane Reynolds
Richard Johnson
7/18/22
x
x
Johnson, Mabel
Jane Reynolds
Richard Johnson
E. Chai, MD
7/18/22
E Chai
 x
Atlantic Heights Rehabilitation Center
718-555-4433
7/18/22
E Chai
E. Chai, MD
x
Click to go back to work-up choices

## Slide 11
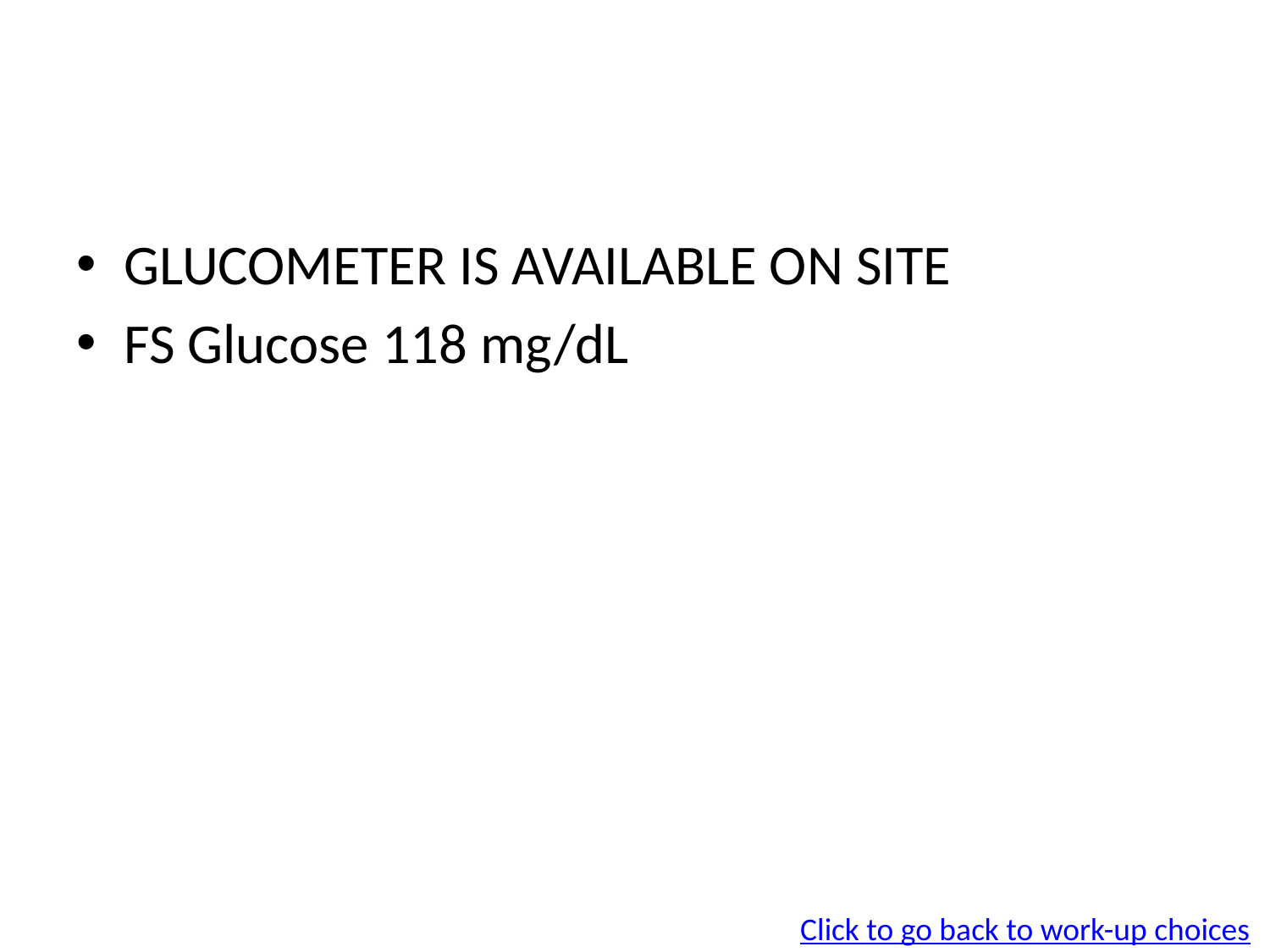

GLUCOMETER IS AVAILABLE ON SITE
FS Glucose 118 mg/dL
Click to go back to work-up choices

## Slide 12
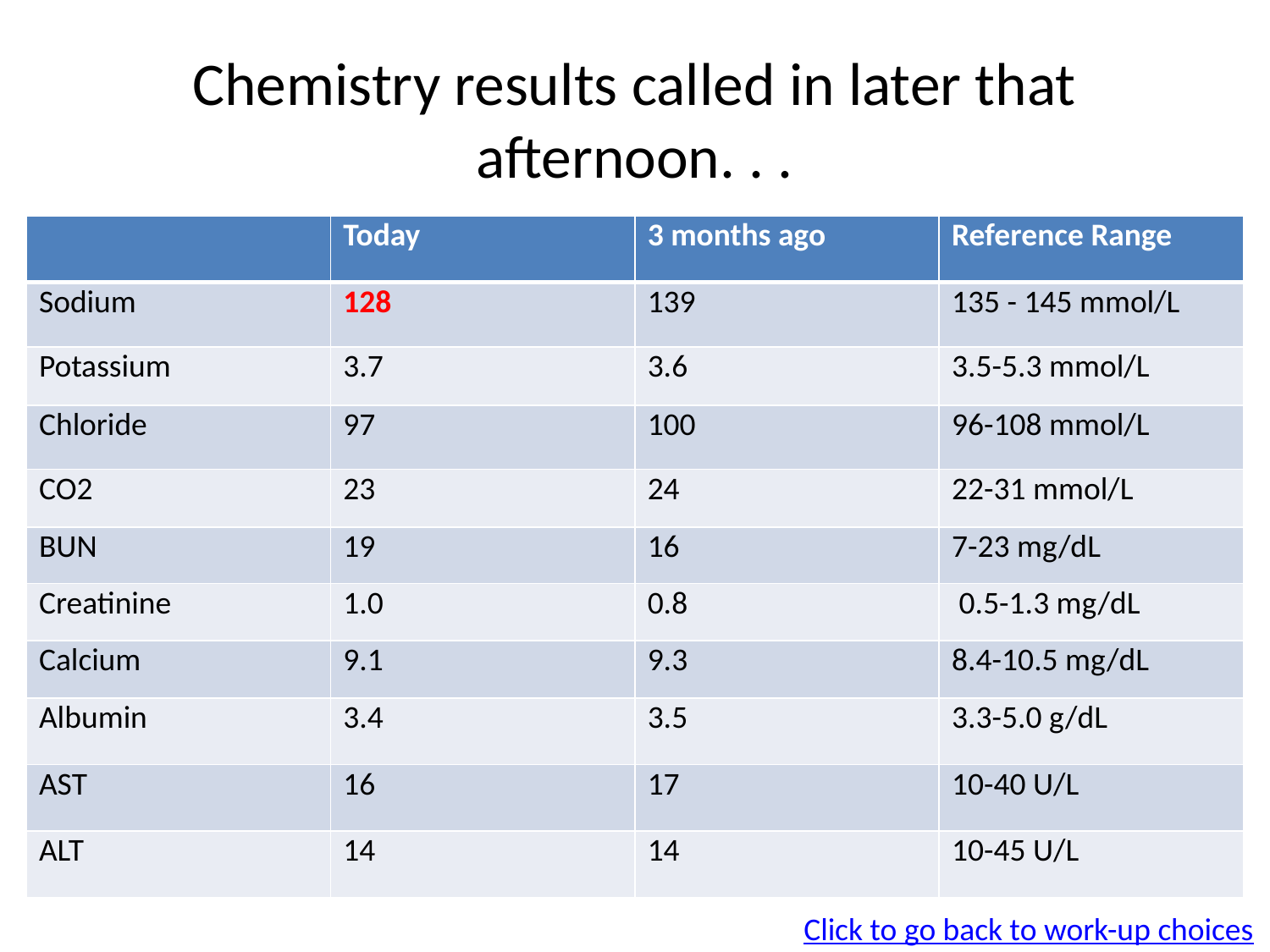

# Chemistry results called in later that afternoon. . .
| | Today | 3 months ago | Reference Range |
| --- | --- | --- | --- |
| Sodium | 128 | 139 | 135 - 145 mmol/L |
| Potassium | 3.7 | 3.6 | 3.5-5.3 mmol/L |
| Chloride | 97 | 100 | 96-108 mmol/L |
| CO2 | 23 | 24 | 22-31 mmol/L |
| BUN | 19 | 16 | 7-23 mg/dL |
| Creatinine | 1.0 | 0.8 | 0.5-1.3 mg/dL |
| Calcium | 9.1 | 9.3 | 8.4-10.5 mg/dL |
| Albumin | 3.4 | 3.5 | 3.3-5.0 g/dL |
| AST | 16 | 17 | 10-40 U/L |
| ALT | 14 | 14 | 10-45 U/L |
Click to go back to work-up choices

## Slide 13
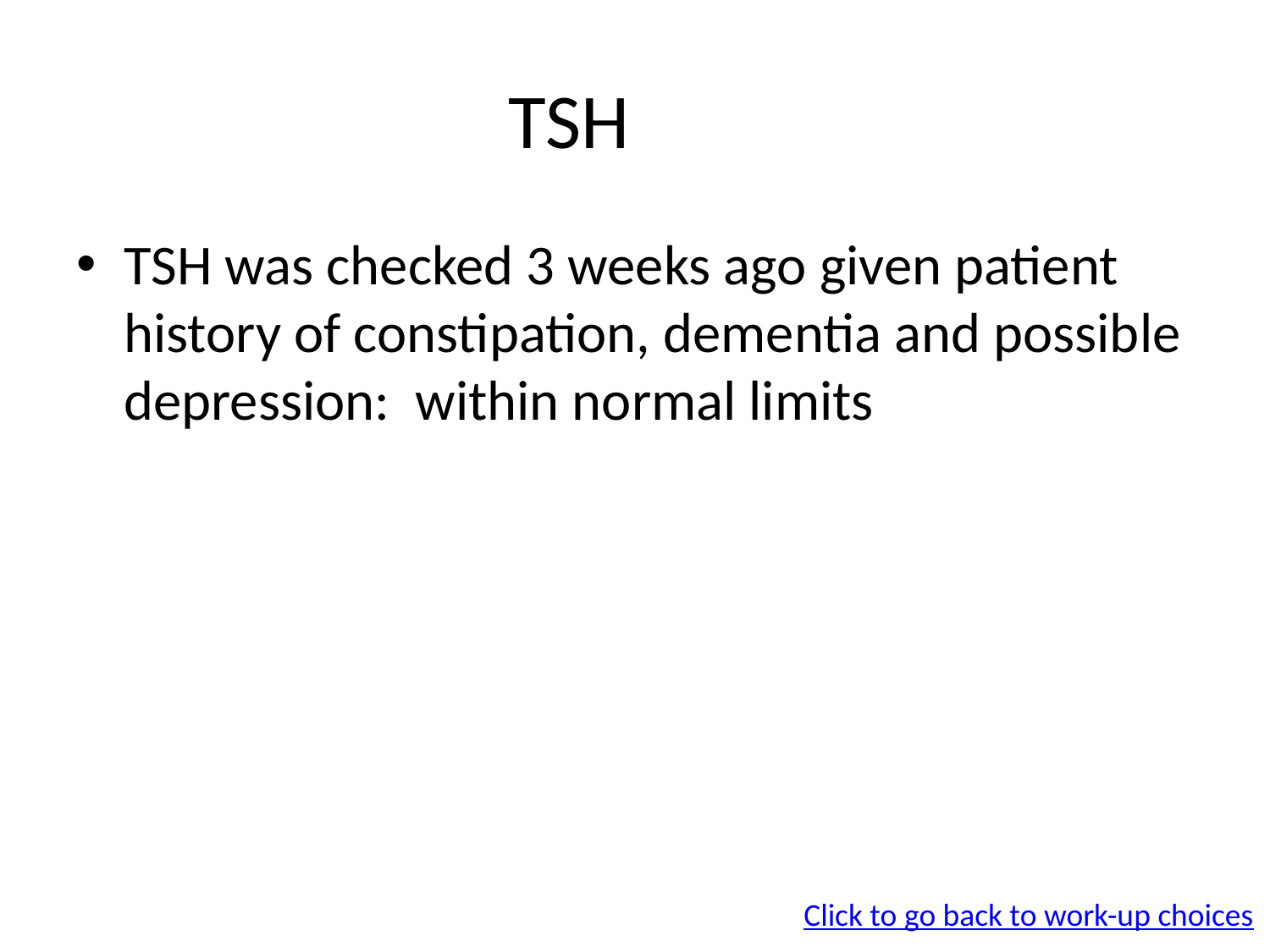

# TSH
TSH was checked 3 weeks ago given patient history of constipation, dementia and possible depression: within normal limits
Click to go back to work-up choices

## Slide 14
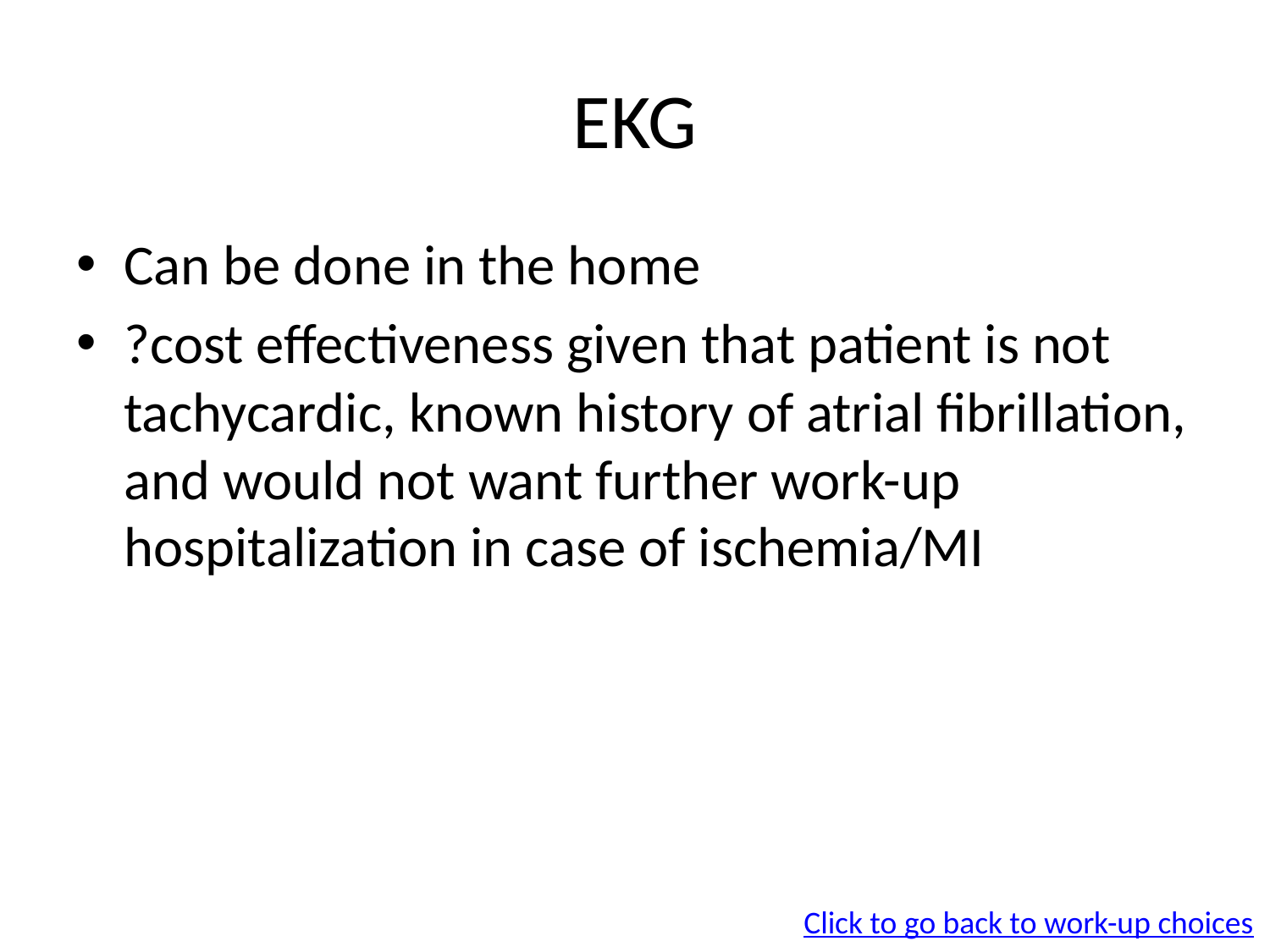

# EKG
Can be done in the home
?cost effectiveness given that patient is not tachycardic, known history of atrial fibrillation, and would not want further work-up hospitalization in case of ischemia/MI
Click to go back to work-up choices

## Slide 15
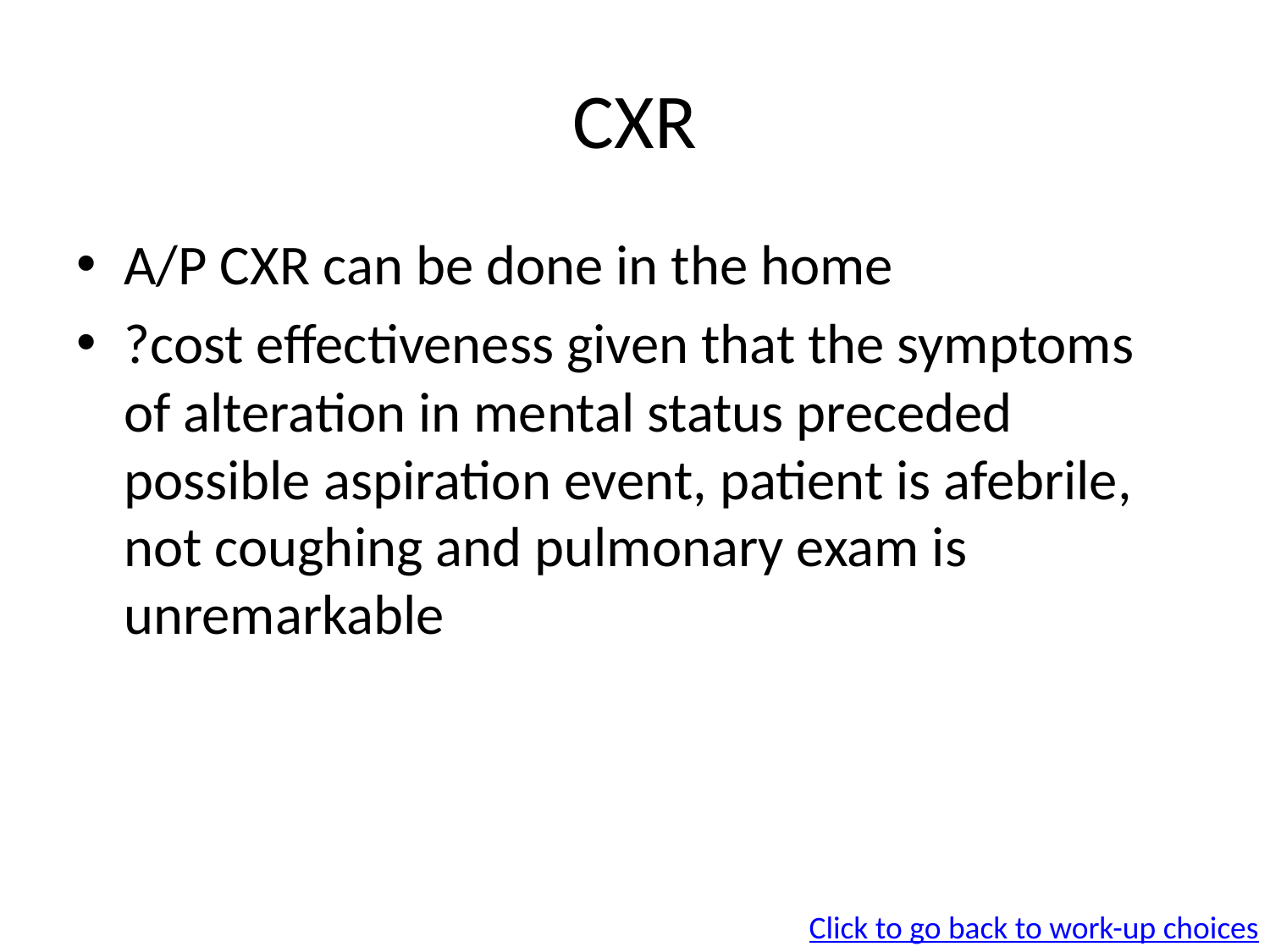

# CXR
A/P CXR can be done in the home
?cost effectiveness given that the symptoms of alteration in mental status preceded possible aspiration event, patient is afebrile, not coughing and pulmonary exam is unremarkable
Click to go back to work-up choices

## Slide 16
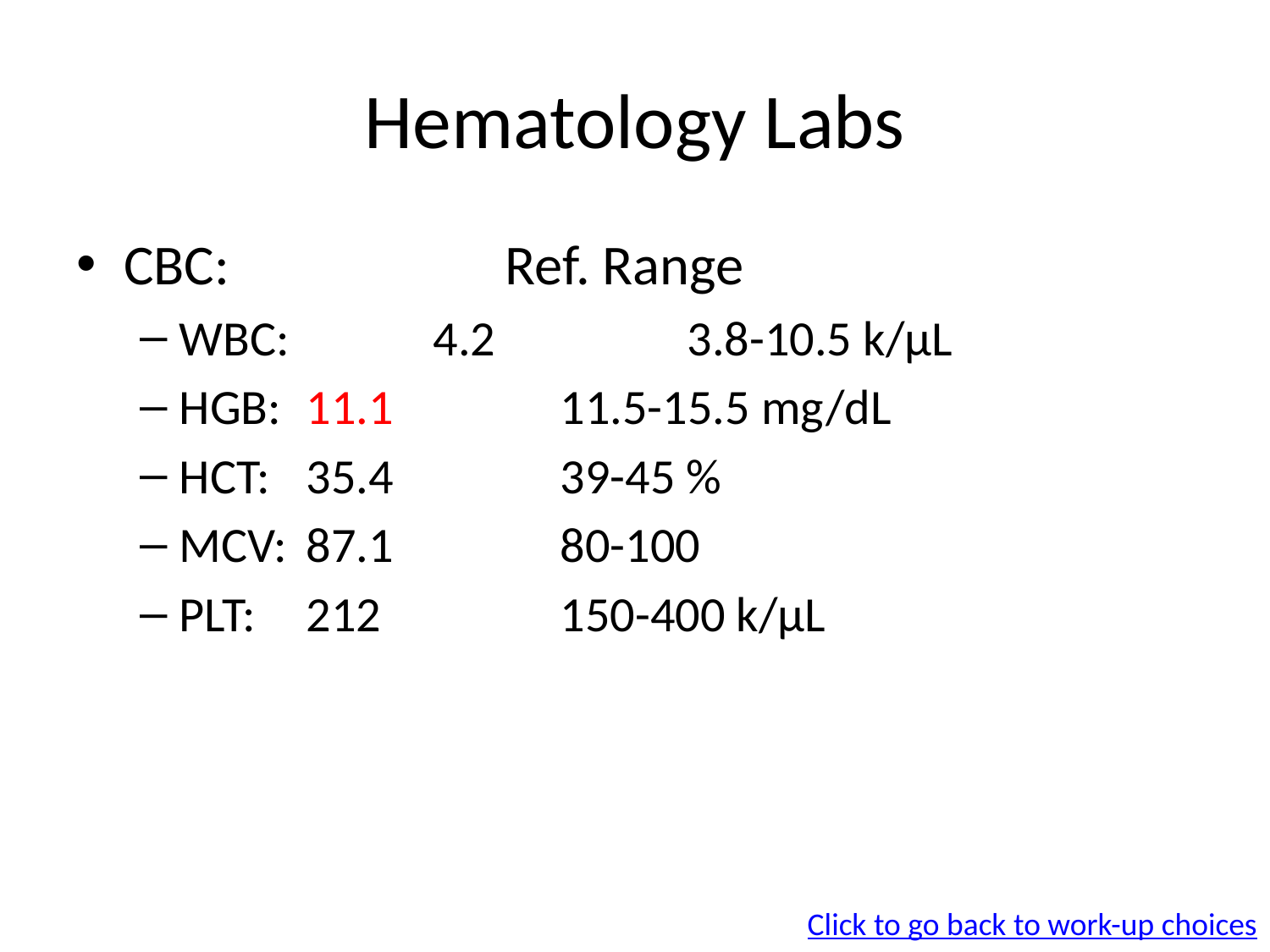

# Hematology Labs
CBC:			Ref. Range
WBC: 	4.2 		3.8-10.5 k/µL
HGB: 	11.1		11.5-15.5 mg/dL
HCT: 	35.4		39-45 %
MCV: 	87.1		80-100
PLT: 	212		150-400 k/µL
Click to go back to work-up choices

## Slide 17
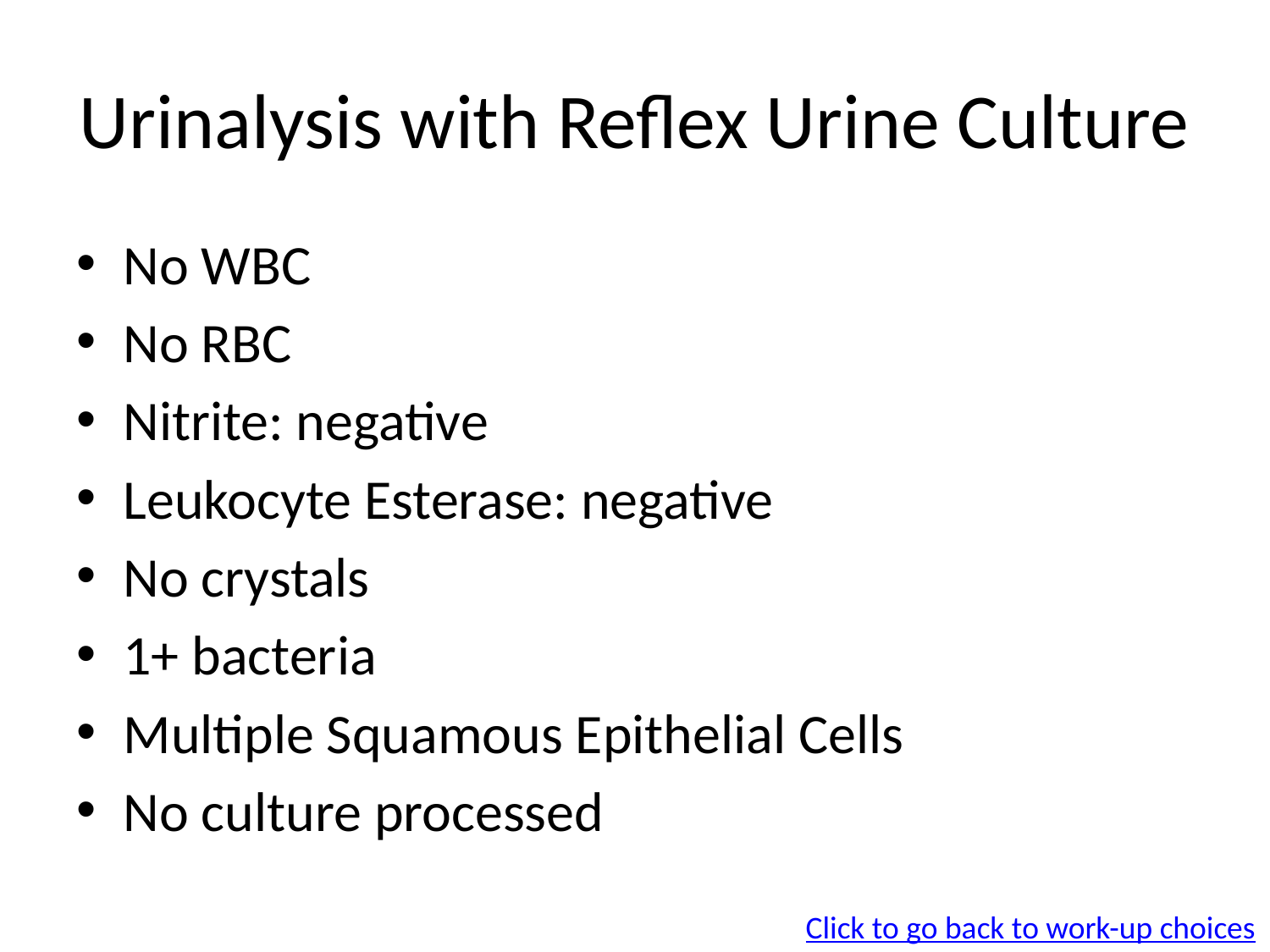

# Urinalysis with Reflex Urine Culture
No WBC
No RBC
Nitrite: negative
Leukocyte Esterase: negative
No crystals
1+ bacteria
Multiple Squamous Epithelial Cells
No culture processed
Click to go back to work-up choices

## Slide 18
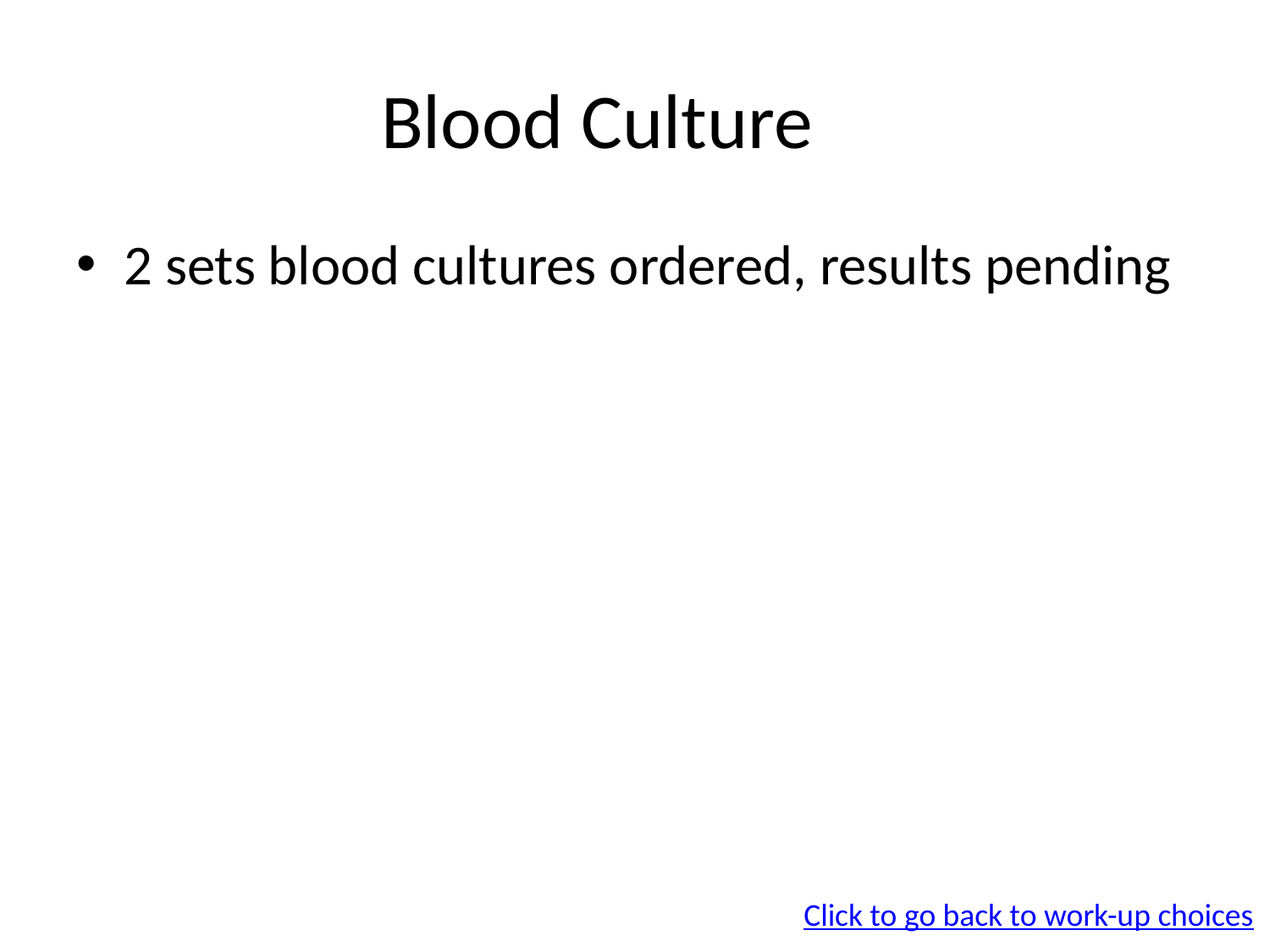

# Blood Culture
2 sets blood cultures ordered, results pending
Click to go back to work-up choices

## Slide 19
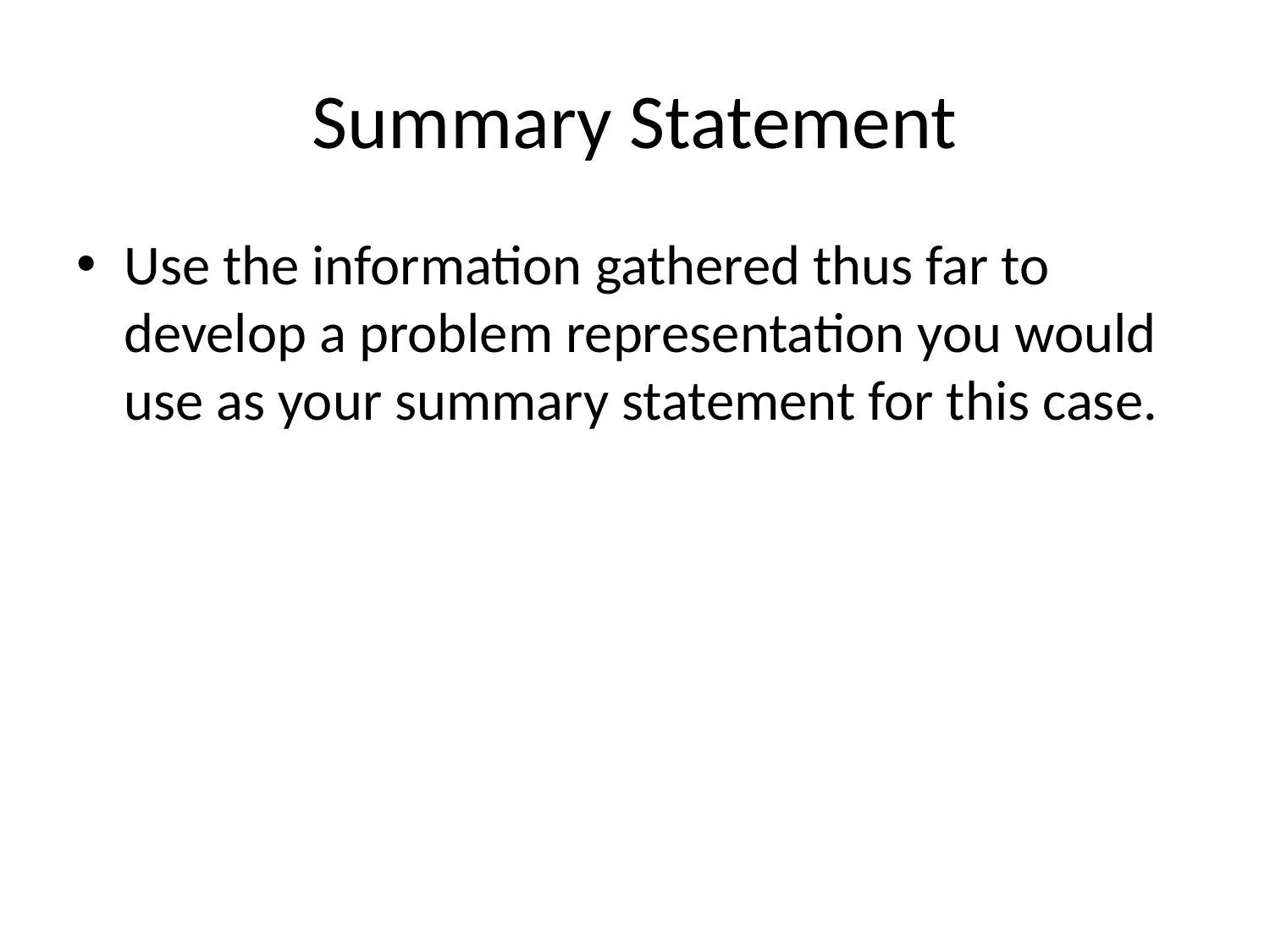

# Summary Statement
Use the information gathered thus far to develop a problem representation you would use as your summary statement for this case.

## Slide 20
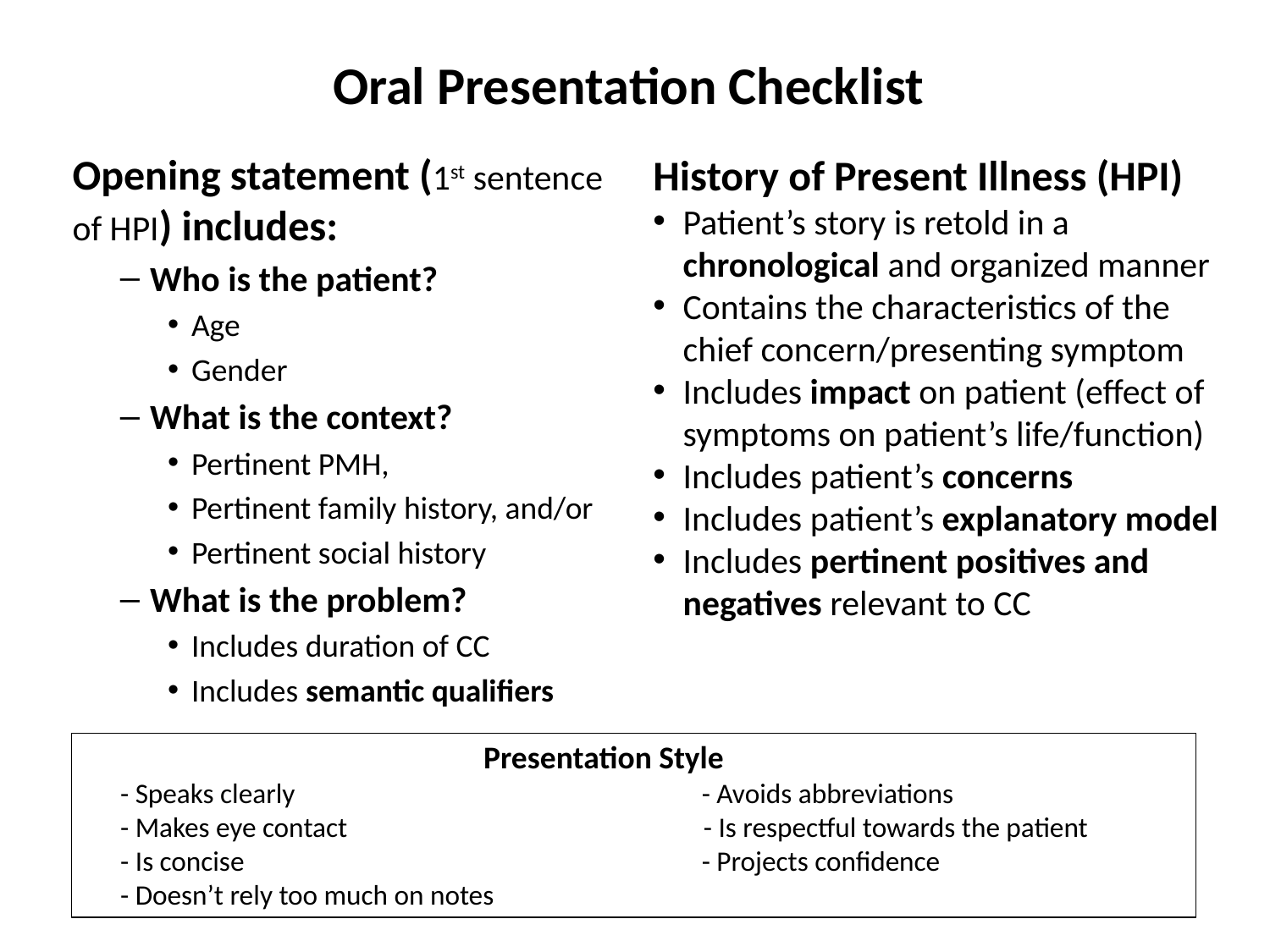

# Oral Presentation Checklist
Opening statement (1st sentence of HPI) includes:
Who is the patient?
Age
Gender
What is the context?
Pertinent PMH,
Pertinent family history, and/or
Pertinent social history
What is the problem?
Includes duration of CC
Includes semantic qualifiers
History of Present Illness (HPI) ​
Patient’s story is retold in a chronological and organized manner​
Contains the characteristics of the chief concern/presenting symptom
Includes impact on patient (effect of symptoms on patient’s life/function)
Includes patient’s concerns
Includes patient’s explanatory model
Includes pertinent positives and negatives relevant to CC​
Presentation Style ​
- Speaks clearly​                                                                - Avoids abbreviations​
- Makes eye contact​                                                        - Is respectful towards the patient​
- Is concise​                                                                        - Projects confidence​
- Doesn’t rely too much on notes
j,gh
