# Supplementary material for: Integration of Geriatrics and Palliative Medicine Into a Medical Student Clinical Reasoning Curriculum
Source: MedEdPORTAL. 2025 Feb 6;21:11495. doi: 10.15766/mep_2374-8265.11495 (PMC11799358; doi:10.15766/mep_2374-8265.11495)
Supplement: Supplementary file 1 — Facilitator Guide.docxPhysical Exam Findings.pptxStudent Survey.docxFaculty Survey.docx [file mep_2374-8265.11495-s001.zip › _Educational Summary Report_11495.pdf]

MedEdPORTAL® | The AAMC Journal of Teaching and Learning Resources

Original Publication Open Access

Integration of Geriatrics and Palliative Medicine Into a Medical Student Clinical Reasoning Curriculum

Julia Caton, MD, EdM, Elaina Suridis, DO, MPH, MS, Gabrielle R. Goldberg, MD, MSEd*

*Corresponding author: gabrielle.goldberg@hofstra.edu

Abstract

Introduction: Integration of geriatrics and palliative medicine principles into preexisting medical student curricula is imperative to train future physicians to care for older adults and those facing serious illness. **Methods:** We developed a case of an older adult presenting with a change in mental status within a preexisting small-group case-based interactive clinical reasoning curriculum. The 1-hour and 50-minute session embedded the 4Ms framework (mentation, medications, mobility, and what matters most) in a clinical case to allow students an organic opportunity to apply the 4Ms in practice while using their communication, clinical reasoning, and hypothesis-driven physical examination skills. Students and faculty completed an end-of-session survey, and each small group's differential diagnoses were reviewed. **Results:** Seventy-five second-year students and 26 faculty participated in the session. On retrospective pre-post surveys, student confidence in all the learning objectives significantly improved. Both students and faculty felt that the integration of geriatrics and palliative medicine was effective. Students valued the topic, appreciated the pedagogical approach and the relevance to clinical preparation, and identified opportunities for continued learning. Students' differential diagnoses demonstrated application of components of three of the four Ms in the 4Ms framework (mentation, medications, and mobility). Notably, many learners did not apply the fourth M (what matters most) to the case without prompting. **Discussion:** This curriculum was well received and effective and can be easily adapted for use with various levels of learners. Faculty should look for additional opportunities to integrate content into preexisting curricular structures.

Keywords
Application, Integration, Clinical Reasoning/Diagnostic Reasoning, Geriatrics, Hospice & Palliative Medicine

Educational Objectives

By the end of this activity, learners will be able to:

1. Apply their communication, clinical reasoning, and hypothesis-driven physical diagnosis skills in the care of an older adult presenting with a change in mental status.
2. Practice gathering a history from a patient's family member.
3. Consider the patient's goals of care in developing a diagnostic and treatment plan for an older adult presenting with a change in mental status.

Introduction

With advances in medical care, people are living longer but often with multiple comorbidities. All physicians must be trained to care for older adults and those living with chronic and life-threatening illnesses. Early integration of geriatrics and palliative medicine principles into medical student education is imperative to train future physicians with the knowledge, skills, and attitudes necessary to care for these vulnerable patients and families.[1] The American Geriatrics Society has published medical school competencies based on Tinetti's 5Ms framework for geriatrics education.[2] The Institute for Healthcare Improvement has adapted this 5Ms framework to a 4Ms framework (mentation, medications, mobility, and what matters most) as the guiding principles for its Age-Friendly Health Systems Initiative to promote evidence-based medical care of older adults.[3] There is considerable overlap in geriatrics and palliative medicine principles and practices, and the 4M framework includes important elements of palliative medicine, particularly what matters most.

Both the 5M and 4M frameworks have been used to develop geriatrics curricula for undergraduate and graduate medical learners. Pedagogical approaches for published curricula designed for medical students include the use of pocket cards

Citation:
Caton J, Suridis E, Goldberg GR. Integration of geriatrics and palliative medicine into a medical student clinical reasoning curriculum. *MedEdPORTAL*. 2025;21:11495.
https://doi.org/10.15766/mep_2374-8265.11495

Copyright © 2025 Caton et al. This is an open-access publication distributed under the terms of the Creative Commons Attribution license.

or geriatrics forms,[4,5] clinical exposure,[6-9] interprofessional workshops,[10,11] and interactive skills-based sessions.[12-14] These curricula have been well received by learners[7,13] and have increased comfort and confidence,[11,12,14] but higher Kirkpatrick levels have not been assessed.[15] Most of these curricula are designed for use in the clinical years. There are few published curricula specifically designed for earlier learners. One of these, a curriculum for second-year medical students, demonstrated a positive impact on learner satisfaction, self-efficacy, and medical knowledge but focuses on only one component of the 4Ms framework: medication management.[11] An immersive curriculum introducing second-year students to different geriatrics models of care was well received and improved student confidence and knowledge; however, this 2-day curriculum requires significant curricular time.[6] A published curriculum from our institution for second-year students based on the 4M framework improved learner confidence, and learners demonstrated the ability to apply skills on a subsequent standardized patient encounter.[16] However, this stand-alone session was not fully integrated into the curriculum.

Despite published geriatrics competencies and geriatrics and palliative medicine curricula, medical students continue to report feeling unprepared to care for seriously ill patients and their families.[17] As many medical schools have shortened or are planning to shorten the preclerkship phase, there are increasing constraints on curricular time.[18] It is therefore necessary to develop opportunities for integration of geriatrics and palliative medicine content into preexisting curricular sessions. The theory of social constructivism, which emphasizes the importance of active integration of new content knowledge into existing cognitive structures within a social context,[19] supports the integration and application of curricular content.

In consideration of the need for increased education in geriatrics and palliative medicine, the effectiveness of prior curricula based on the 4Ms framework, limitations on curricular time, and the theory of social constructivism, we developed a case-based, small-group session integrating geriatrics and palliative medicine content into our preexisting clinical reasoning curriculum (Clinical Learning Sessions [CLSs]). This novel curriculum is the first based on the 4M framework that makes use of social constructivist theory to integrate geriatrics and palliative medicine content knowledge in a preexisting clinical reasoning curriculum. We hypothesized that students would use their preexisting knowledge of the 4Ms in their approach to the case and that, after participating in the curriculum, students would report increased confidence in their ability to apply clinical skills in the care of an older adult and to consider a patient's goals of care in developing a management plan.

Methods

The session's aim was to give learners an opportunity to apply previously learned geriatrics and palliative medicine concepts to a clinical case. The target audience for this session was second-year students, but the session could be used with any learner group with prior knowledge of geriatrics and palliative medicine concepts. Students needed the skills necessary to gather a history, develop a differential diagnosis for a patient with a change in mental status, and plan a hypothesis-driven physical examination. Small groups consisted of seven or eight students with two facilitators per group. Faculty participating in the session had to be comfortable facilitating learner-centered small-group sessions.

Curricular Context

The Donald and Barbara Zucker School of Medicine at Hofstra/Northwell (ZSOM) employed an integrated, case/problem-based curriculum during the first 2 years. The first- and second-year courses integrated basic science, anatomy, clinical manifestations of disease, and clinical experiences. All ZSOM courses also featured health-system sciences sessions, including a geriatrics and palliative medicine curricular thread. This thread began in the first year with a patient-centered communication curriculum, an introduction to palliative medicine session, and a session on advance care planning. The final course of the second year covered neurobiology, the nervous system structure, neurology, and psychiatry. This course included components of the geriatrics and palliative medicine thread, including dedicated sessions on living with dementia and an introduction to geriatrics.[16]

Clinical Learning Faculty

CLS faculty represented a wide range of clinical backgrounds, including internal medicine, pediatrics, and emergency medicine. All faculty received an orientation to the curriculum with a focus on developing comfort and skill in facilitating student-directed learning. A 30-minute faculty development meeting was held the morning of each session to address any questions about case content, discuss challenges and successes from prior sessions, and allow faculty partners to chat together in advance of the start of the session.

CLS Curriculum

CLSs started in the first year. The curriculum was originally developed in 2016 based on a previously published curriculum at

Copyright © 2025 Caton et al. This is an open-access publication distributed under the terms of the Creative Commons Attribution license.

the Geisel School of Medicine at Dartmouth.[20] In the Dartmouth curriculum, students were provided the patient case in written format. In contrast, our CLSs required learners to elicit the patient history from a faculty member portraying the patient. This approach promoted authenticity as it allowed students to apply their communication skills and medical knowledge to think broadly as they would in a patient encounter. CLSs occurred three times per course. Students met in groups of seven to eight students with two faculty facilitators. Cases were developed considering where students were in the curriculum, such that they could generate a reasonable differential diagnosis around the patient's chief concern. One week before a session, faculty received the faculty guide (Appendix A) and PowerPoint (Appendix B). The faculty guide contained detailed case notes to allow one faculty to prepare for the patient's role.

Sessions were 1 hour and 50 minutes. Students did not have access to the case topic or learning objectives prior to the session. During the first hour of the session, students elicited a history from the faculty member portraying the patient. The faculty guide (Appendix A) instructed faculty on the facilitation of the session such that students took turns gathering the history, with frequent time-outs to allow for reflection and debrief on communication skills and discussion of diagnostic hypotheses. At each pause, the faculty member who was not playing the role of the patient prompted the student gathering the history to reflect on their communication skills. Faculty and peers provided additional reinforcing feedback. The first time-out took place immediately after elicitation of the chief concern, to allow students to think broadly. The group used the whiteboards in the classroom to organize and document their diagnostic hypotheses. Faculty facilitated the discussion using Socratic questions to ensure students used an organized approach to generate a wide differential. Students took turns gathering additional pieces of the patient's history. With each pause in the history gathering, students added, edited, and reprioritized their diagnostic hypotheses. Faculty encouraged students to use their knowledge of the illness scripts of specific diagnoses on their differential to guide their history gathering. After the history gathering, students narrowed their hypotheses to two or three leading and "do not miss" diagnoses. Students then broke up into smaller groups of two or three and discussed their plan for the hypothesis-driven physical examination. After students had regrouped and compared their plans, faculty shared a PowerPoint presentation (Appendix B), which depicted the patient's physical examination and relevant laboratory or radiographic findings. Faculty facilitated student discussion and interpretation of these data.

CLS Case: Change in Mental Status in an Older Adult

The final case in the CLS curriculum was an older adult patient presenting with their adult child for a home visit due to a change in mental status. The ultimate diagnosis was hyponatremia from syndrome of inappropriate antidiuretic hormone secretion secondary to sertraline. The students had learned about the pathophysiology of hyponatremia during their first year. Their current course included sessions on delirium, dementia, and an introduction to geriatrics based on the 4Ms framework, all of which occurred prior to this CLS. The students were approaching their first inpatient clerkships, during which they would be caring for many older adults facing serious illness. We chose to integrate geriatrics and palliative medicine content in this final CLS case in consideration of social constructivist theory, which supported application of recently learned content in real-world contexts in service of deeper learning.[19]

The session followed the standard CLS format as described above. A novel addition to the session was the inclusion of hyperlinks in the PowerPoint allowing the students to select the next step in management from a list of options and be taken to a slide depicting the result of selecting that management option. If students selected an option that would require transfer to the hospital, the link took them to the patient's Medical Orders for Life-Sustaining Treatment (MOLST) form (Appendix B), which specified that the patient did not wish to be hospitalized. Facilitators made certain that students viewed the results of all workup options in the slide set before the session ended, ensuring that all students reviewed and considered the patient's wishes documented on the MOLST.

Evaluation

At the conclusion of the session, we invited students and faculty to complete an exit survey. The survey was administered on paper to allow for completion in real time and to maximize the response rate. The student survey (Appendix C) consisted of five retrospective pre-post questions assessing confidence in carrying out the educational objectives of the session, one Likert-style question to assess student agreement with the effectiveness of integrating geriatrics and palliative medicine concepts into the case, and an open-ended question eliciting students' thoughts on the integration of the MOLST form into the case. We chose to use a retrospective pre-post design as the objectives were not explicitly shared with learners at the beginning of the session and to allow matching of student data without gathering any identifying information, thereby maintaining participant anonymity and confidentiality. The faculty survey (Appendix D) consisted of five questions eliciting faculty's assessment of learners' ability to

Copyright © 2025 Caton et al. This is an open-access publication distributed under the terms of the Creative Commons Attribution license.

accomplish the objectives during the session, one Likert-style question to assess the effectiveness of integrating geriatrics and palliative medicine concepts into the case for student application of prior knowledge, and an open-ended question eliciting faculty thoughts on the integration of the MOLST form into the case.

During the session, students used whiteboards to brainstorm their diagnostic hypotheses. Photos were taken of the differential diagnoses generated on the whiteboards in each of the small-group rooms. The photos were reviewed for type of organizational system used, inclusion of 4Ms content in the differential diagnosis, and inclusion of the ultimate diagnosis of selective serotonin reuptake inhibitor–induced hyponatremia.

The Hofstra University Institutional Review Board deemed this project exempt from ethical review.

Statistical Analysis

All data from both surveys were manually transferred to an Excel database. We used IBM SPSS Statistics software (version 28.0) for statistical analysis. Descriptive statistics for ordinal variables were presented as the median and interquartile range. Wilcoxon signed rank tests were used to compare student confidence (5-point Likert scale: 1 = *not confident*, 5 = *very confident*) relating to the educational objectives before and after the session. We conducted a thematic analysis on the narrative responses from the student survey. Each of us independently reviewed and coded student responses to the final open-ended survey question. We used an iterative and inductive approach to identify codes and reach consensus on codes, themes, and supporting quotes.[21]

Results

Seventy-five (75%) second-year students and 26 faculty participated in the session. Due to lower-than-expected attendance because of multiple students with viral illness on the day of the session, two of the original 13 small groups were combined, forming 12 small groups with six to eight learners per group. Seventy-three students completed the student survey; one student did not consent to have their responses included in research and their responses were excluded from analysis (response rate = 96%). Although we did not complete the faculty survey, the 23 remaining faculty did (response rate = 100%).

Student Surveys

There was a statistically significant improvement in student confidence (Kirkpatrick level 1) in students' ability to perform all educational objectives (Table and Figure 1). All 73 students agreed or strongly agreed with the statement "I found the integration of geriatrics and palliative medicine concepts into today's CLS case an effective means of applying prior knowledge" (Kirkpatrick level 1).

The student exit survey asked the students to "share your thoughts, observations, and/or reactions to the integration of the patient's MOLST form in the evaluation and management discussion of today's CLS case." The codes and themes generated based on student responses and representative quotations are shown in Figure 2.

Students' Approach to Diagnostic Hypotheses

Most of the groups (10) used a mechanistic organizational approach to the differential diagnosis, employing the VINDICATE mnemonic with the addition of a neuro and/or psych category (vascular, infection/inflammation, neoplasm, drugs/degenerative, iatrogenic/idiopathic, congenital, autoimmune/allergy, trauma, endocrine/environmental, neurologic/psychiatric). One group used a body system approach, and one group used a hybrid approach. All groups applied elements of the 4Ms framework to their diagnostic hypotheses (Kirkpatrick level 3). Eleven groups (92%) included falls and/or complications of falls, 11 (92%) included memory and/or mood diagnoses, 12 (100%) included medication side effects and/or polypharmacy, and seven (58%) included electrolyte abnormalities or hyponatremia in their differential.

Table. Retrospective Pre- and Postsession Student Confidence Ratings ($N = 72$)

	Mdn (IQR)		
Item[a]	Pre	Post	Test Statistics
Student confidence in:			
Ability to apply their communication skills in the care of an older adult patient presenting with a change in mental status	3 (3-4)	4 (4-4)	$z = -6.144$, $p < .001$
Ability to apply their clinical reasoning skills in the care of an older adult patient presenting with a change in mental status	3 (3-3)	4 (3-4)	$z = -5.552$, $p < .001$
Ability to apply their hypothesis-driven physical exam skills in the care of an older adult patient presenting with a change in mental status	3 (3-4)	4 (3-4)	$z = -6.171$, $p < .001$
Ability to gather a history from a patient's family member	4 (3-4)	4 (4-5)	$z = -5.154$, $p < .001$
Ability to consider a patient's goals of care in developing a diagnostic and treatment plan for an older adult patient presenting with a change in mental status	3 (2-4)	4 (3-4)	$z = -5.384$, $p < .001$

Abbreviation: IQR, interquartile range.
[a]Rated on a 5-point Likert scale (1 = *not confident*, 2 = *slightly confident*, 3 = *moderately confident*, 4 = *confident*, 5 = *very confident*).

Copyright © 2025 Caton et al. This is an open-access publication distributed under the terms of the Creative Commons Attribution license.

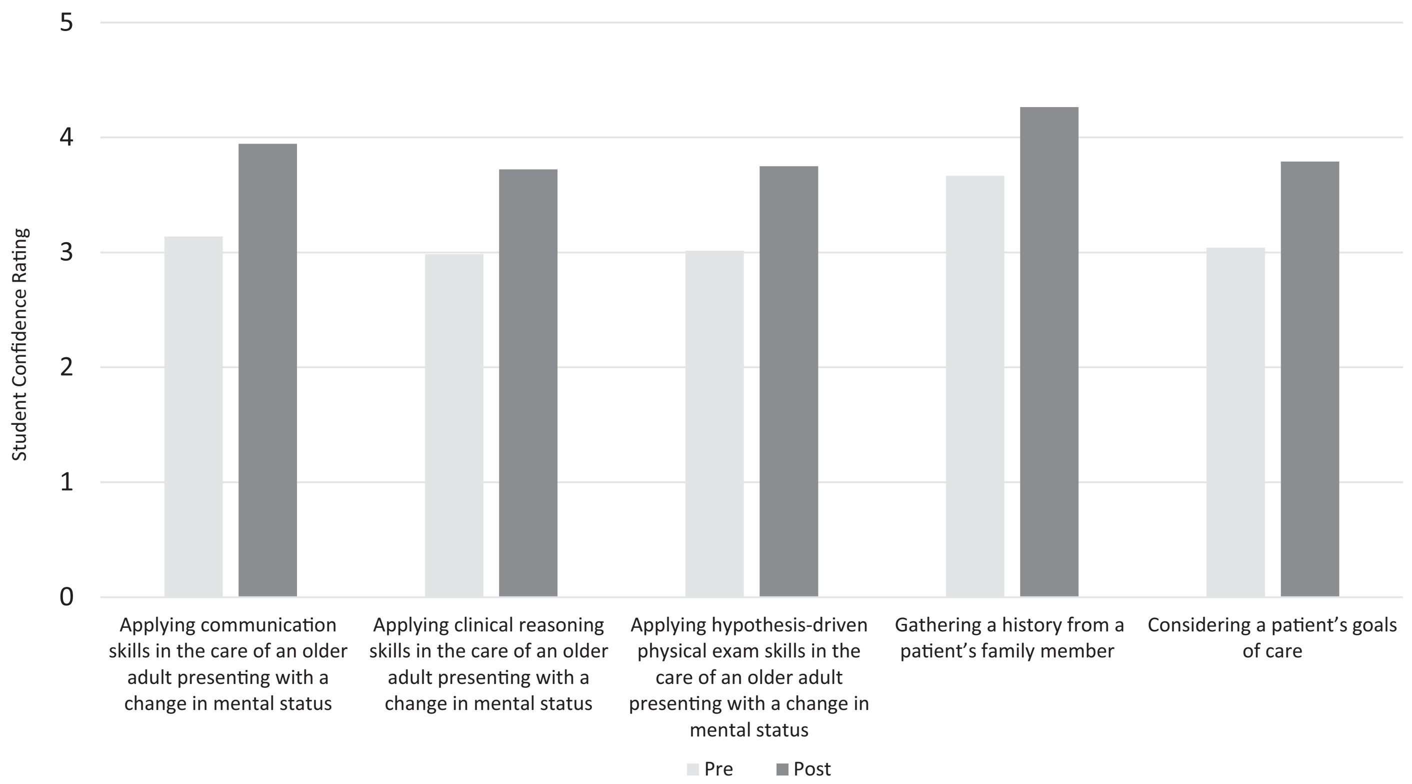

Figure 1. Retrospective pre- versus postsession student confidence ratings on a 5-point Likert scale (1 = *not confident,* 2 = *slightly confident,* 3 = *moderately confident,* 4 = *confident,* 5 = *very confident*). $N = 72$. For all comparisons, $p < .001$.

Faculty Surveys

The faculty rated how effectively learners accomplished the session learning objectives (Figure 3). The majority of faculty felt learners were able to apply their communication skills (96%), clinical reasoning skills (96%), and physical diagnosis skills (78%) and gather a history from a family member (100%) very effectively or extremely effectively in the care of an older adult patient presenting with a change in mental status (Kirkpatrick level 2). Fifty-seven percent of faculty felt learners could very effectively or extremely effectively consider a patient's goals of care in developing a diagnostic and treatment plan for an older adult presenting with a change in mental status. The remaining faculty felt students were able to do so somewhat effectively.

All faculty agreed or strongly agreed with the statement "I found the integration of geriatrics and palliative medicine concepts into today's CLS case an effective means of allowing students to apply prior knowledge" (Kirkpatrick level 2). In narrative feedback, faculty shared that the session was an important integration of content but noted that students did not discuss goals of care prior to proposing a plan for diagnostic evaluation.

Discussion

This session effectively integrated geriatrics and palliative medicine educational objectives into a preexisting clinical reasoning curriculum. The session was well received by students, who valued the topic and appreciated the pedagogical approach of integration and application; improved students' confidence; and allowed students to apply the 4Ms framework to their differential diagnostic consideration. The integration of these concepts into a preexisting clinical reasoning curriculum allowed students to collaborate, apply content, and problem-solve in a novel real-world scenario consistent with social constructivist theory.[19] An additional benefit was that integration of this content into an existing curricular structure required no additional curricular time.

Review of student whiteboard writing generated during the session revealed that students consistently applied three (mentation, mobility, and medication) of the 4Ms in generating their diagnostic hypotheses, an encouraging sign that they were able to engage with and apply prior knowledge. However, both students and faculty observed that there was little or no discussion or consideration of the patient's goals (the fourth M,

Copyright © 2025 Caton et al. This is an open-access publication distributed under the terms of the Creative Commons Attribution license.

Themes (Frequency)	Codes (Frequency)	Representative Comments
Value of topic (37)	Reminder/exposure (16)	• "I honestly wouldn't have jumped to asking about health care goals until the very end of the work-up when would require transport to the hospital so I def became aware of asking about this earlier and the importance of this."<br>• "I thought that seeing what the MOLST form looks like and what information it contains was very helpful."
	Important topic (8)	
	More time for MOLST (7)	
	Helpful (6)	
Pedagogical approach (27)	Integration (11)	• "I liked the integration of the form into the case. I liked the links to the various tests so we can order different labs. It was important to remember patient preference and desires in their care through the MOLST."<br>• "Much better way to discuss concepts of palliative/geriatrics than large group session. Good case that felt real-life. Good psych integration. Allowed for basic science conversation. MOLST form not discussed. Excellent use of [PowerPoint] for PE findings."
	Application (9)	
	Spiraling to past courses/course content (4)	
	Use of hyperlinks/ interactive (3)	
Clinical preparation (20)	Patient-centered (9)	• "Made the case very real—this is what we'll see on rotation, so exposure is important."<br>• "Very important given our clinical rotations starting soon. I also really enjoyed the labs/imaging being hyperlinked, it made it more fun."
	Realistic (8)	
	Preparation for clinical care (3)	
Emotional responses (15)	Enjoyable (7)	• "I really appreciated it because I did not think to consider it, quite honestly. So I am glad to have had that brought to my attention."<br>• "I loved it! I think it's very important to include this in more healthcare conversations. My grandmother had one and it definitely needs to be represented more in med education. "
	Appreciation/gratitude (6)	
	Frustrating (2)	
Opportunities for continued learning (12)	Did not consider goals until end (6)	• "None of us anticipated the MOLST form before seeing it. It seemed like an effective way to drive these forms home."<br>• "I wish we had more time to discuss this because I am curious to learn more about MOLST forms + older adults."
	Prompted future consideration (3)	
	Uncertainty about next steps (3)	

Figure 2. Thematic analysis of student exit surveys. Abbreviations: MOLST, Medical Orders for Life-Sustaining Treatment; PE, physical exam.

what matters most) until the workup section of the case, when they were explicitly prompted by the appearance of the patient's MOLST form. This was consistent with what has been observed with experienced practicing clinicians.[22] Older adults, particularly those with multiple comorbidities, want to make decisions for themselves pertaining to their goals of care.[23] Discussion of what matters most must therefore take place as an intrinsic part of an older adult's medical care, starting with the gathering of the patient's history.

Based on the results of the student and faculty surveys, the social constructivist approach to integrating the MOLST form into this case was an effective means of underscoring the importance of consideration of advance care planning. However, a major limitation of our curricular evaluation is that it was not designed to measure the impact of this experience on students' clinical practice. Another important limitation is that we did not have access to an appropriate comparison group for our survey, so opted to use a retrospective pre-post survey design as the next best alternative. We chose this approach because it is thought to be more accurate than a traditional pre-post design. However, it could have introduced bias into our results as respondents may have tended to report that learning took place regardless of whether it had or not.[24] Furthermore, this educational session was a onetime event at a single institution. Future directions to better evaluate this activity would be to obtain true baseline presession data and to gather data from multiple institutions using the curriculum. An additional future direction would be to obtain more robust postsession data with longer-term follow-up to assess whether this educational experience translates into

Copyright © 2025 Caton et al. This is an open-access publication distributed under the terms of the Creative Commons Attribution license.

MedEdPORTAL® The AAMC Journal of Teaching and Learning Resources

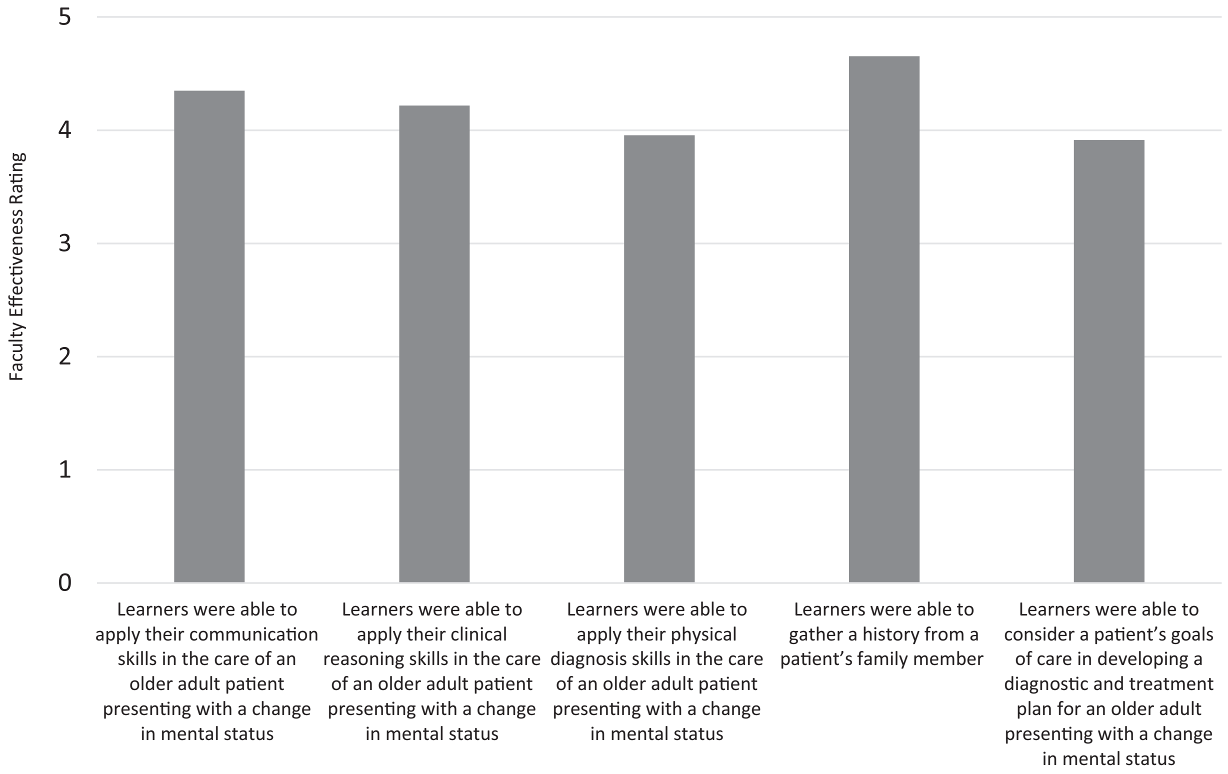

Figure 3. Faculty assessment of how effectively the learning objectives were accomplished as rated on a 5-point Likert scale (1 = *not at all effectively,* 2 = *a little bit effectively,* 3 = *somewhat effectively,* 4 = *very effectively,* 5 = *extremely effectively*). *N* = 23.

students' future consideration of their patients' goals of care earlier in the illness trajectory, perhaps using assessments from clerkship-level faculty. While we had high faculty and student survey response rates, a final limitation is that student attendance at this CLS was lower than expected, which may have contributed to survey nonresponse bias.

This session was developed for use with second-year students within a preexisting clinical reasoning curriculum but has applicability to many different learners. The case and interactive PowerPoint can be adapted for use with a larger student group in educational settings with limitations on facilitator availability. The case and faculty guide provided in this curriculum can be easily adapted to different levels of medical school learners, residency-level learners, and other health professions students, providing an opportunity to integrate and apply geriatrics and palliative medicine content knowledge.

Appendices

A. Facilitator Guide.docx

B. Physical Exam Findings.pptx

C. Student Survey.docx

D. Faculty Survey.docx

All appendices are peer reviewed as integral parts of the Original Publication.

Julia Caton, MD, EdM: Assistant Professor, Division of Hospital Medicine, Department of Medicine, Northwell Health and Donald and Barbara Zucker School of Medicine at Hofstra/Northwell; ORCID: https://orcid.org/0000-0001-5687-8902

Elaina Suridis, DO, MPH, MS: Attending Physician, Division of Geriatrics and Palliative Medicine, Department of Medicine, Northwell Health; ORCID: https://orcid.org/0009-0009-2805-1401

Gabrielle R. Goldberg, MD, MSEd: Associate Professor and Director, Clinical Skills, Department of Science Education, Donald and Barbara Zucker School of Medicine at Hofstra/Northwell; ORCID: https://orcid.org/0000-0001-7837-7041

Disclosures

None to report.

Copyright © 2025 Caton et al. This is an open-access publication distributed under the terms of the Creative Commons Attribution license.

Funding/Support
None to report.

Ethical Approval
The Hofstra University Institutional Review Board deemed further review of this project not necessary.

References

1. Meiboom AA, de Vries H, Hertogh CMPM, Scheele F. Why medical students do not choose a career in geriatrics: a systematic review. *BMC Med Educ*. 2015;15:101. https://doi.org/10.1186/s12909-015-0384-4
2. *Minimum Competencies in Geriatrics for Medical Students*. AGS Geriatrics Healthcare Professionals; date unknown. Accessed January 14, 2025. https://adgap.americangeriatrics.org/sites/default/files/media/files/AGS%20Geriatric%205Ms%20Competencies%20for%20Medical%20Students.pdf
3. Age-friendly health systems. Institute for Healthcare Improvement. Accessed January 14, 2025. https://www.ihi.org/initiatives/age-friendly-health-systems
4. Golden A, Berry A, Cannarozzi ML, Dangiolo MB, UCF Medical Student Comprehensive Geriatric Assessment. *MedEdPORTAL*. 2012;8:9251. https://doi.org/10.15766/mep_2374-8265.9251
5. Holliday AM, Hawley CE, Schwartz AW. Geriatrics 5Ms pocket card for medical and dental students. *J Am Geriatr Soc*. 2019;67(12):E7-E9. https://doi.org/10.1111/jgs.16226
6. Byerly LK, Rivera J. Geri Models of Care (MOC): an immersive preclerkship curriculum fostering student exploration of residential geriatric models of care. *MedEdPORTAL*. 2022;18:11262. https://doi.org/10.15766/mep_2374-8265.11262
7. Weissman D. Palliative care experience for 3rd year medical students. *MedEdPORTAL*. 2006;2:164. https://doi.org/10.15766/mep_2374-8265.164
8. Goldlist K, Beltran CP, Rhodes-Kropf J, Sullivan AM, Schwartz AW. Out of the classroom, into the home: medical and dental students' lessons learned from a geriatrics home visit. *J Am Geriatr Soc*. 2022;70(9):2659-2665. https://doi.org/10.1111/jgs.17968
9. Byerly LK, Floren LC, Yukawa M. Fostering interprofessional geriatric patient care skills for health professions students through a nursing facility–based immersion rotation. *MedEdPORTAL*. 2020;16:11059. https://doi.org/10.15766/mep_2374-8265.11059
10. Larson C, O'Brien B, Rennke S. GeriWard Falls: an interprofessional team-based curriculum on falls in the hospitalized older adult. *MedEdPORTAL*. 2016;12:10410. https://doi.org/10.15766/mep_2374-8265.10410
11. Hawley CE, Triantafylidis LK, Phillips SC, Schwartz AW. Brown bag simulation to improve medication management in older adults. *MedEdPORTAL*. 2019;15:10857. https://doi.org/10.15766/mep_2374-8265.10857
12. Wilson LA, Harder B, Kelley C, Powell R, Foster M, Roberts E. Geriatric telehealth: a standardized patient case for medical students. *MedEdPORTAL*. 2023;19:11345. https://doi.org/10.15766/mep_2374-8265.11345
13. Talwalkar JS, Fortin AH, Morrison LJ, et al. An advanced communication skills workshop using standardized patients for senior medical students. *MedEdPORTAL*. 2021;17:11163. https://doi.org/10.15766/mep_2374-8265.11163
14. Blazek M. Geriatric psychiatry: just so you know. *MedEdPORTAL*. 2014;10:9722. https://doi.org/10.15766/mep_2374-8265.9722
15. Kirkpatrick JD, Kirkpatrick WK. *Kirkpatrick's Four Levels of Training Evaluation*. ATD Press; 2016.
16. Goldberg GR, Solis G, John JT, Olvet DM, Kranz KA. 4Ms for early learners: a skills-based geriatrics curriculum for second-year medical students. *MedEdPORTAL*. 2022;18:11264. https://doi.org/10.15766/mep_2374-8265.11264
17. Reidy JA, Brizzi K, Chan SH, et al. Curricular reform in serious illness communication and palliative care: using medical students' voices to guide change. *Acad Med*. 2024;99(5):550-557. https://doi.org/10.1097/ACM.0000000000005647
18. Verma N, Yui JC, Record JD, Hueppchen NA, Naik RP. The changing landscape of the preclinical medical school curriculum: results from a nationwide survey of United States medical school curriculum deans. *Am J Med*. 2024;137(2):178-184.e2. https://doi.org/10.1016/j.amjmed.2023.10.021
19. Vygotsky LS. *Mind in Society: The Development of Higher Psychological Processes*. Harvard University Press; 1978.
20. Weinstein A, Pinto-Powell R. Introductory clinical reasoning curriculum. *MedEdPORTAL*. 2016;12:10370. https://doi.org/10.15766/mep_2374-8265.10370
21. Kiger ME, Varpio L. Thematic analysis of qualitative data: AMEE Guide no. 131. *Med Teach*. 2020;42(8):846-854. https://doi.org/10.1080/0142159X.2020.1755030
22. Jimenez G, Tan WS, Virk AK, Low CK, Car J, Ho AHY. Overview of systematic reviews of advance care planning: summary of evidence and global lessons. *J Pain Symptom Manage*. 2018;56(3):436-459.e25. https://doi.org/10.1016/j.jpainsymman.2018.05.016
23. González-González AI, Schmucker C, Nothacker J, et al. End-of-life care preferences of older patients with multimorbidity: a mixed methods systematic review. *J Clin Med*. 2020;10(1):91. https://doi.org/10.3390/jcm10010091
24. Chang R, Little TD. Innovations for evaluation research: multiform protocols, visual analog scaling, and the retrospective pretest–posttest design. *Eval Health Prof*. 2018;41(2):246-269. https://doi.org/10.1177/0163278718759396

Received: July 29, 2024
Accepted: December 13, 2024
Published: February 6, 2025

Copyright © 2025 Caton et al. This is an open-access publication distributed under the terms of the Creative Commons Attribution license.
